# Functional MRI correlates of emotion regulation in major depressive disorder related to depressive disease load measured over nine years

**DOI:** 10.1016/j.nicl.2023.103535

**Published:** 2023-11-11

**Authors:** Rozemarijn S. van Kleef, Amke Müller, Laura S. van Velzen, Janna Marie Bas-Hoogendam, Nic J.A. van der Wee, Lianne Schmaal, Dick J. Veltman, Maria M. Rive, Henricus G. Ruhé, Jan-Bernard C. Marsman, Marie-José van Tol

**Affiliations:** a Department of Biomedical Sciences of Cells and Systems, Cognitive Neuroscience Center, University Medical Center Groningen, Groningen, the Netherlands; b Department of Psychology, Helmut Schmidt University / University of the Federal Armed Forces Hamburg, Hamburg, Germany; c Orygen Parkville, VIC, Centre for Youth Mental Health, University of Melbourne, Melbourne, VIC, Australia; d Developmental and Educational Psychology, Institute of Psychology, Leiden University, Leiden, the Netherlands; e Department of Psychiatry, Leiden University Medical Center, Leiden, the Netherlands; f Leiden Institute for Brain and Cognition, Leiden University Medical Center, the Netherlands; g Department of Psychiatry, Amsterdam UMC location VUMC & Amsterdam Neuroscience, Amsterdam, the Netherlands; h Department of Psychiatry, Amsterdam UMC location AMC, Amsterdam, the Netherlands; i Triversum, Department of Child and Adolescent Psychiatry, GGZ Noord-Holland Noord, Hoorn, the Netherlands; j Department of Psychiatry, Radboudumc, Nijmegen, the Netherlands; k Donders Institute for Brain, Cognition and Behavior, Radboud University, Nijmegen, the Netherlands

**Keywords:** Depression, Emotion regulation, Disease load, Functional connectivity, Brain activity

## Abstract

Major Depressive Disorder (MDD) often is a recurrent and chronic disorder. We investigated the neurocognitive underpinnings of the incremental risk for poor disease course by exploring relations between enduring depression and brain functioning during regulation of negative and positive emotions using cognitive reappraisal.

We used fMRI-data from the longitudinal Netherlands Study of Depression and Anxiety acquired during an emotion regulation task in 77 individuals with MDD. Task-related brain activity was related to disease load, calculated from presence and severity of depression in the preceding nine years. Additionally, we explored task related brain-connectivity. Brain functioning in individuals with MDD was further compared to 35 controls to explore overlap between load-effects and general effects related to MDD history/presence.

Disease load was not associated with changes in *affect* or with brain *activity,* but with *connectivity* between areas essential for processing, integrating and regulating emotional information during downregulation of negative emotions. Results did not overlap with general MDD-effects. Instead, MDD was generally associated with lower parietal activity during downregulation of negative emotions. During upregulation of positive emotions, disease load was related to connectivity between limbic regions (although driven by symptomatic state), and connectivity between frontal, insular and thalamic regions was lower in MDD (vs controls).

Results suggest that previous depressive load relates to brain connectivity in relevant networks during downregulation of negative emotions. These abnormalities do not overlap with disease-general abnormalities and could foster an incremental vulnerability to recurrence or chronicity of MDD. Therefore, optimizing emotion regulation is a promising therapeutic target for improving long-term MDD course.

## 1 Introduction

Major Depressive Disorder (MDD) is one of the most prevalent psychiatric disorders, often characterized by a chronic or recurrent course (Hardeveld et al., 2013. 2013., Eaton et al., 2008, Burcusa and Iacono, 2007, Burley, 2016, Verhoeven et al., 2018). Around 30 % of individuals with MDD do not reach clinical remission within one year (Boland and Keller, 2002, Keller, 1982) and relapse rates up to 50 % within two years after remission have been reported (Richards, 2011, Kanai et al., 2003). Relapse and chronicity rates increase with more severe, recurrent, and persistent symptomatology (Underwood et al., 2018. 2018., Kessing et al., 2004, Lewinsohn et al., 1989). This cumulative burden (or ‘disease load’) may be associated with alterations in (neuro)cognitive and affective processing (Van Rijsbergen et al., 2015. 2015., Vrijsen et al., 2014), facilitating unfavourable course of MDD. Understanding the neurocognitive effects of depressive disease load is important for understanding individual disorder trajectories.

It has been proposed that vulnerability to MDD is characterized by biased processing of negative information, facilitating negative affect, and by an inability to effectively regulate affective states. This may result in sustained periods of sad mood, ultimately spiralling into a depression (Teasdale, 1988, Nolen-Hoeksema et al., 2008, Gotlib, 2010, Ochsner and Gross, 2005, Holtzheimer and Mayberg, 2011). Dysfunctional emotion regulation may not only be related to developing a depressive state, but also to perpetuation of symptoms and recurrence of depressive episodes (Berking et al., 2014, ten Doesschate et al., 2010). Studying emotion regulation in relation to disease load may thus be highly relevant for understanding mechanisms underlying depressive course and thereby for altering its long-term course.

In general, emotion regulation has been associated with functioning of brain regions involved in cognitive control (e.g., the dorsolateral prefrontal cortex (PFC), somatosensory area and parietal cortex), primary emotion processing (e.g., the amygdala) and areas involved in salience processing and appraisal (e.g., the anterior insula, ventrolateral PFC, and anterior cingulate cortex) (Buhle et al., 2014, Frank et al., 2014, Kohn et al., 2014). In addition to activity within specific areas, connectivity in frontoparietal ‘executive’ and cingulo-opercular ‘salience’ brain networks, associated with cognitive control and the integration of sensory, emotional, and cognitive information, respectively, have been found to underpin emotion regulation (Banks et al., 2007, Morawetz et al., 2016, Morawetz et al., 2017). In MDD, abnormalities in activity and connectivity of areas in these networks during emotion regulation have consistently been observed, including abnormal activity and connectivity in lateral frontal, parietal and superior temporal areas, the insula, and the amygdala (Rive et al., 2013, Zilverstand et al., 2017, Picó-Pérez et al., 2017, Kaiser et al., 2015. 2015.). Some studies suggest that these emotion-regulation abnormalities are state-independent characteristics, persisting upon remission (Kanske et al., 2012, Smoski et al., 2015, Dong et al., 2019) and contributing to vulnerability to recurrent episodes (ten Doesschate et al., 2010). However, other studies failed to corroborate these findings (Rive et al., 2015, Van Kleef et al., 2022, Wolkenstein et al., 2014).

So far, there is some evidence suggesting that enduring depression may be accompanied by progressive changes in brain structure and function of regions underpinning the regulatory control of emotions. For example, duration of depressive episodes has been associated with hippocampal atrophy (Sheline et al., 2013, Colla et al., 2007, Schmaal et al., 2016) and seed-based amygdala resting state connectivity (Jin et al., 2011), suggesting a cumulative ‘scarring’ effect. However, other neuroimaging studies reported no relation between depressive duration (e.g., years since onset of depression or total time spent with depression) or previous depressive burden and grey matter volume (Binnewies et al., 2021, van Tol et al., 2010. 2010.). Functionally, time spent with depression, measured retrospectively over a two-year interval, was not related to brain activity during an emotional encoding task (Ai et al., 2020), though symptomatology over this two-year period could be predicted from brain activity during emotional processing (Ai et al., 2020, Frässle et al., 2020, Opmeer et al., 2016, Schmaal et al., 2015) in the same study sample. Importantly, the effects of cumulative depressive burden or disease load on the neural underpinnings of emotion regulation, and thus the potential increasing role of emotion regulation abnormalities in the accumulating risk of persistence or recurrence, are until now unexplored. Most studies so far have merely focused on years since onset of depression, number of episodes, or time spent with depressive symptoms. These approaches address important aspects related to longitudinal course, but there’s no study yet focusing on how the cumulation of presence and severity of depressive symptoms (i.e., cumulative depressive load) characterized over a long period, relate to the neurocognitive underpinnings of emotion regulation in MDD.

Here we aimed to explore the relationship between disease load and brain functioning during instructed emotion regulation using cognitive reappraisal vs. attendance. To this end, we used unique data from the Netherlands Study of Anxiety and Depression (NESDA), for which individuals with MDD were followed prospectively over a nine-year period. NESDA provided the opportunity to measure both duration and burden as a combined clinical proxy of disease load prospectively over nine years and relate this to brain functioning during emotion regulation measured at the nine-year follow-up. We studied brain activity during downregulation of negative as well as upregulation of positive emotions, as inadequate upregulation of positive effects may additively underpin the vulnerability to depressive symptomatology (Gradin et al., 2011, Heller et al., 2009, Light et al., 2011. 2011.). Next, we explored connectivity of brain regions within networks that seem relevant for understanding MDD (Banks et al., 2007, Kaiser et al., 2015. 2015., Morawetz et al., 2016) and emotion regulation (Kohn et al., 2014, Rive et al., 2013, Zilverstand et al., 2017, Picó-Pérez et al., 2017, Frank et al., 2014), namely frontoparietal and cingulo-opercular networks. We examined both activity and connectivity as these measures represent different characteristics of brain functioning. Here, activity may inform on the net-outcome of regional brain involvement, whereas connectivity informs on a network aspect detailing how regional brain activity is coordinated across the brain. We hypothesized lowered activity and connectivity of brain areas within frontoparietal and cingulo-opercular networks during regulation of negative and positive emotions as a function of higher disease load. To examine whether load-related alterations represent a worsening of the abnormalities generally observed in MDD instead of constituting an autonomous vulnerability to unfavourable course, we additionally explored whether load-dependent changes overlapped with MDD-related abnormalities. To this end, we compared activity and connectivity patterns of individuals with MDD to control participants who never suffered from a psychiatric disorder.

## 2 Materials and methods

### 2.1 Participants

For the current analysis, functional Magnetic Resonance Imaging (fMRI)-data of the nine-year follow-up of the Netherlands Study of Depression and Anxiety (NESDA) study were used, in addition to clinical information regarding disease course collected over six measurements in the preceding nine years. NESDA is a longitudinal observational cohort study on the course of depression and anxiety disorders, which took place in three centres (VU Medical Center Amsterdam, Leiden University Medical Center and University Medical Center Groningen, all located in the Netherlands). The protocol of the NESDA study has been described elsewhere (Penninx et al., 2008, Penninx et al., 2021). The ethical review committees of the three participating centres approved this study and all participants provided written informed consent.

At baseline (Wave 1 (W1)), the NESDA study included data from 2981 individuals, who were followed up after one year (W2), two years (W3), four years (W4), six years (W5), and nine years (W6). Of the 2981 baseline participants, 301 respondents participated in a neuroimaging ancillary study at W1. A full description of the longitudinal NESDA neuroimaging study is provided in (van Tol et al., 2021).

In the present study, data were included from the W6 neuroimaging measurement, including 95 individuals with MDD. For this measurement, all individuals who participated in the W1 neuroimaging study and at that time fulfilled criteria for an MDD diagnosis in the past six months, were invited to participate. Additionally, participants with MDD from the full NESDA W6 sample were invited to participate in the W6 neuroimaging measurement if they fulfilled criteria for a one-month diagnosis of MDD at the W6 interview. Thus, participants with MDD fulfilled criteria for an MDD diagnosis in the past 9 years (W1-W6) and were currently (at W6) either remitted or depressed. Diagnoses were established with the structured Composite International Diagnostic Interview (CIDI) (Robins et al., 1988).

To explore whether load relations showed spatial overlap with general MDD-associated neural abnormalities relative to never-depressed controls (NDC). We additionally included data from 41 NDC, who had no lifetime history of psychiatric illness. We screened data from the Life Chart Interview (LCI; see below) for MDD and excluded those who reported one or more months with any depressive core symptom.

### 2.2 Measures

#### 2.2.1 Diagnostic characteristics

The CIDI interview was conducted at each consecutive NESDA wave by trained research staff. Other clinical state characteristics were determined with the Inventory of Depressive Symptomatology (IDS) (Rush et al. , 2009, Rush et al., 1996) and the Beck Anxiety Inventory (BAI) (Beck et al., 1988) at W6.

If participants scored positive on one of the two core symptoms (depressed mood or anhedonia) of the CIDI MDD-module in any month over the measured nine years, the Life Chart Interview (LCI) (Lyketsos et al., 1994) was conducted. The LCI provides a graphical overview of a period of time, including participants’ recalled life events to aid one’s memory, through which the presence and severity (experienced burden) of depressive symptoms per month could be assessed. Using this interview, an overview of the fluctuation of depressive symptoms over time was determined. This instrument has good psychometric properties (Warshaw et al., 1994).

Cumulative disease load of depression over nine years was calculated based on the LCI, administered at W3 (3-year FU since BL), W4, W5, and W6, covering all months between each consecutive measurement. To weigh presence of depressive symptoms with severity of these symptoms over nine years, we calculated the average symptom severity (on a scale from 0 to 4) per month between W1 and W6, a method proven valid for assessing the severity of depressive mood fluctuations in bipolar disorder (Denicoff et al., 2000). We recoded LCI severity scores to 0 (=no severity or no symptoms), 0.25 (low severity), 0.5 (moderate severity), 0.75 (high severity) and 1 (very high severity). When participants reported absence of the two core symptoms of MDD (depressed mood or anhedonia), that month was recoded as 0. We summed these scores and divided them by the total number of months between W1 and W6 (in line with (Denicoff et al., 2000).

#### 2.2.2 Emotion regulation task

An Emotion Regulation Task (based on previous studies (Johnstone et al., 2007, Rive et al., 2015, Van der Velde et al., 2015. 2015., Van Kleef et al., 2022) was performed during blood-oxygen-level-dependent (BOLD) imaging. The task consisted of five conditions: three ‘attend’ conditions and two ‘regulate’ conditions. During the attend conditions, participants were instructed to passively attend to negative, positive, or neutral emotional images. During the two regulate conditions, they were instructed to either downregulate their emotional response to negative emotional images, or to upregulate their emotional response to positive emotional images, using cognitive reappraisal techniques. Images were selected from the International Affective Picture System (IAPS) (Lang et al., 2008) and images presented during the attend and regulate conditions for the positive and negative valences were matched on valence, arousal, dominance, and type of visual scenery (see Supplement 1). Before participants went into the scanner, the instructions (“attend”, “downregulate”, and “upregulate”) were trained until satisfactory performance was achieved, using images not used in the actual MRI-task.

**Fig. 1:**
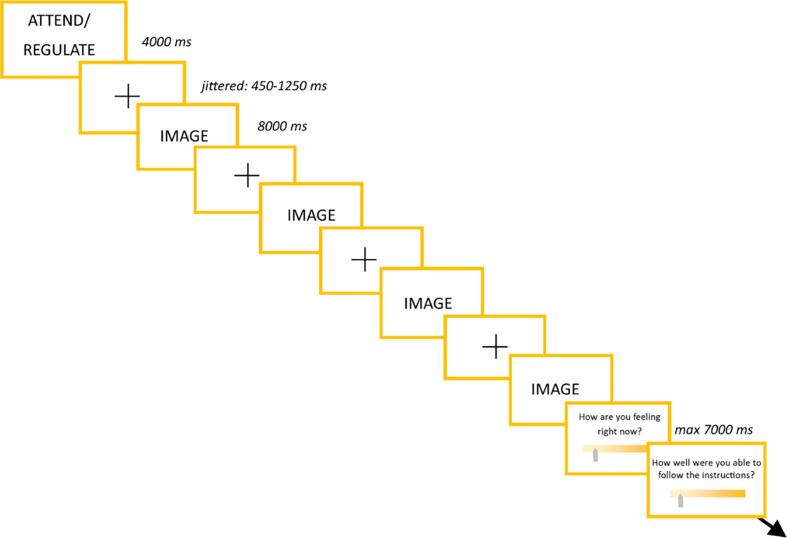
*Emotion Regulation Task. Note.* Example of the order of presentation during one experimental block. The instruction (“attend” or “regulate”) was presented, followed by four IAPS-pictures of the same valence, and two VAS ratings. The instruction and pictures were separated with fixation jitters lasting between 450 and 1250 ms, consisting of a “+”. In total, one block took approximately 50 s and each block was separated by a jittered fixating, varying from eight to 14.5 s.

Each condition comprised three blocks with four images presented in each block (in pseudo-randomized order) (See Fig. 1). The task started and ended with a neutral attend block. Each image was presented for eight seconds and separated by a jittered fixation cross. After each block, participants were instructed to report how they were feeling (ranging from “very sad” to “very happy”) and how well they were able to follow the instructions during that block (ranging from “not at all” to “very well”) on a visual analogue scale (VAS), ranging from 0 to 100. After response to these questions (maximum of seven seconds to respond), the block finished with a jittered fixation cross, with varying durations between eight and 14.5 s. In total, fifteen blocks were presented in a pseudorandomized order, comprising a total of 60 pictures, equally divided over the five conditions. The total task duration was approximately 16 min.

### 2.3 Image acquisition

MRI data were collected with 3 T Philips MR-scanners at the three participating centers (Spinoza Centre for Neuroimaging Amsterdam, Leiden University Medical Center, and University Medical Center Groningen), all using a 32-channel head coil.

High-resolution anatomical T1-weighted images were obtained (TR = 9.0 ms, TE = 3.5 ms, 170 sagittal slices, voxel size 1x1x1mm), and used in the present study for reorientation purposes. Functional imaging sensitive for the BOLD-effect were acquired using an EPI sequence (TR = 2000 ms, TE = 30 ms, 37 transverse slices acquired in descending order, voxel size 2.5x2.5x2.7 mm).

### 2.4 Behavioural data analyses

#### 2.4.1 Demographic, clinical, and behavioural characteristics

Demographic-, clinical-, and behavioural data were analysed with SPSS25 (SPSS Inc., Chicago, IL, USA). To assess distribution of demographic and clinical characteristics over the MDD and NDC participants, we performed non-parametric (because normality assumptions were violated) Mann-Whitney U tests on age, sex, educational level, IDS scores, and BAI scores.

#### 2.4.2 Affect responsivity in relation to disease load

The relation between self-reported affect during the Emotion Regulation Task with load was assessed by calculating bivariate Spearman correlations between disease load and the VAS affect ratings for the five task conditions (‘attending negative images’, ‘attending positive images’, ‘attending neutral images’, ‘downregulating negative images’, and ‘upregulating positive images’) in the MDD group. Additionally, we explored the relation of load with the difference between the affective ratings following the regulate blocks vs the attend blocks (regulate – attend) for the negative and positive conditions, separately.

#### 2.4.3 Affect responsivity generally associated with MDD

Additionally, general MDD-related abnormalities in affect responsivity during the emotion regulation task were assessed by setting up non-parametric Mann-Whitney U tests for the five task conditions, with group (MDD vs NDC) as between-subjects factor. Effects were considered significant at the level of *α* = 0.05 (two-sided). Alpha was corrected for the number of performed tests by using the Simple Interactive Statistical Analysis Bonferroni tool (SISA Bonferroni; https://www.quantitativeskills.com/sisa/calculations/bonfer.htm). Additionally, we explored the differences between MDD and NDC on the difference in affective ratings following the regulate blocks vs the attend blocks was calculated (regulate – attend) for the negative and positive conditions, separately.

### 2.5 FMRI data analyses

#### 2.5.1 FMRI pre-processing

FMRI data were processed using SPM12 (v7487; Welcome Trust Centre for Neuroimaging, London, UK), implemented in Matlab 8.5.0 (R2015a; Mathworks, Natick, MA, USA)). A standard pre-processing pipeline was used, including manual reorientation to the anterior-posterior commissure plane, realignment, co-registration (structural to functional mean image), two-step normalisation of functional images to MNI via the co-registered structural image, spatial smoothing (8 mm full-width half-maximum Gaussian kernel). Furthermore, to control for excessive motion, framewise displacement was calculated. Volumes were flagged if exceeding a threshold of 0.9 mm, to be regressed out during subject level modelling (in line with (Siegel et al., 2014). See the Supplement 2 for the pre-processing script.

#### 2.5.2 FMRI subject-level modelling

On the subject-level, pre-processed data were modelled within the framework of the general linear model (SPM12). The event-related model included regressors for the onsets and duration of five task conditions (within condition, onset and duration of each picture was modelled), instructions and affect and performance ratings, volumes marked as motion (based on framewise displacement; one regressor per volume), and time and dispersion derivatives, and convolved with a canonical hemodynamic response function (HRF). Contrast images were created based on the beta-estimates reflecting peak-height for downregulation of negative emotions (‘downregulating vs attending negative images’), and for upregulation of positive emotions (‘upregulating vs attending positive images’).

#### 2.5.3 FMRI group-level modelling

For all group-level analyses, the SNPM toolbox was used, a toolbox within SPM12 designed for non-parametric permutation tests, based on a generalized linear modelling approach (Nichols and Holmes, 2001); https://warwick.ac.uk/snpm). A non-parametric approach was chosen to avoid the risk of false positive findings related to multiple comparison correction relying on parametric assumptions (Eklund et al., 2016, Han et al., 2019). Furthermore, for all group level analyses described below (i.e., all peak activation and gPPI analyses), we included sex, age, education level, scanner site (two dummies), and motion (mean Framewise Displacement)as covariates of no interest.

#### 2.5.4 BOLD responses during emotion regulation in relation to disease load

Within the MDD sample, we set up two regression models for assessing peak activity of the BOLD response: one including disease load as regressor and the ‘downregulating vs attending negative images’-contrast images as dependent variable, and one including disease load as regressor and the ‘upregulating vs attending positive images’-contrast images as dependent variable. We additionally ran these models with current depressive episode at W6 (yes/no), IDS and BAI scores at time of W6 scanning, and antidepressant medication use (yes/no) at W6 as additional covariates to correct for possible effects of clinical characteristics.

#### 2.5.5 Functional connectivity during emotion regulation in relation to disease load

To perform task-related connectivity analyses, we used generalized Psychophysiological Interaction (gPPI) modelling, using the gPPI toolbox implemented in SPM12 (McLaren et al., 2012). We created 6 mm spheres around left and right peak coordinates from main task effects and selected those coordinates that were in accordance with previous emotion regulation studies (Zilverstand et al., 2017, Picó-Pérez et al., 2017, Buhle et al., 2014). Thus, we selected six bilateral regions, including the (left and right) amygdala, dorsal anterior cingulate cortex (dACC), dorsolateral PFC (DLPFC), ventrolateral PFC (VLPFC), supplementary motor area (SMA) and superior parietal cortex. See Supplement 3 for more information on these seeds. For each participant a gPPI model was estimated for each seed region. Following hemodynamic deconvolution, the time series were multiplied by the task regressors and convolved with the HRF. Functional connectivity was calculated between the time courses of the seed regions and all other voxels in the brain for the ‘downregulating vs attending negative images’ and ‘upregulating vs attending positive images’ contrasts.

To assess the relationship between disease load and functional connectivity patterns during emotion regulation, we set up regression models per gPPI seed and per valence within the SNPM toolbox, including disease load as regressor, and subject-specific gPPI maps for the contrasts ‘downregulating vs attending negative images’ and ‘upregulating vs attending positive images’ as dependent variable. We additionally ran these models with current depressive episode at W6 (yes/no), IDS and BAI scores at W6, and antidepressant medication use (yes/no) at W6 as additional covariates to correct for possible effects of clinical state and medication (see Supplement 9).

#### 2.5.6 Brain activity and connectivity generally associated with MDD

To assess emotion regulation abnormalities generally observed in MDD relative to NDC, we set up a two-sample *t*-test per valence (‘downregulating vs attending negative images’ and ‘upregulating vs attending positive images’), including group (MDD vs NDC) as between-group factor and the contrast images created at subject-level as dependent variables.

For the connectivity analyses, non-parametric two-sample *t*-test models per gPPI seed and per valence were set up, including group (MDD vs NDC) as between-group factor, and the coupling between the subject-level gPPI’s for the contrasts ‘downregulating vs attending negative images’ and ‘upregulating vs attending positive images’ as dependent variables.

#### 2.5.7 Statistical thresholding

Cluster-based inference was based on a pre-set significance level of *α* < 0.05, family-wise error rate corrected for multiple comparisons, based on non-parametric permutation testing (5000 permutations) with the cluster forming threshold set at 3.09. Given the explorative nature of the study and the interdependence of the seed-based connectivity maps (because of the voxels’ intercorrelations), we did not additionally correct for the number of seed-based functional connectivity tests (Streiner and Norman, 2011).

## 3 Results

### 3.1 Demographic and clinical characteristics

Initially, 95 participants were included in the MDD sample. Eighteen participants were excluded because of missing data or poor data quality (due to excessive motion or scanner-related artefacts), leading to a sample of 77 for the MDD-group. Twenty-eight participants fulfilled criteria for a major depressive episode in the last month. The number of months between W1 and W6 in which participants in the MDD group reported presence of MDD symptomatology ranged from one until 109 months (*M = 52.51, SD = 30.50*). For further demographic and clinical information, and the distribution of disease load (*M* = 0.48, *SD* = 0.13) in individuals within the MDD group see Supplement 4.

For assessment of disease-general abnormalities in comparison to controls, 41 participants were included in the NDC group. Of these participants, six were excluded because of missing data or poor data quality. The final NDC group consisted of 35 participants. See Supplement 4 for demographic characteristics of the NDC group and a comparison of these characteristics to the MDD group.

### 3.2 Behavioural results

#### 3.2.1 Main task effects

Across groups, behavioural main task effects revealed higher affect scores after the two positive task conditions (*M =* 67.14, *SD =* 12.80*, p* < 0.001), and lower affect scores after the two negative task conditions (*M =* 39.83, *SD =* 14.44*, p* < 0.001), compared to neutral (*M =* 57.20, *SD =* 9.16).

Across groups, a main task effect of instruction was found: affect scores were higher after the ‘downregulating negative images’ and ‘upregulating positive images’ conditions (*M =* 55.09, *SD =* 9.67) than after the ‘attending negative images’ and ‘attending positive images’ conditions (*M =* 51.88, *SD =* 9.45, *p =* 0.013).

#### 3.2.2 Affect responsivity and disease load in MDD

Within the MDD group, cumulative disease load over the past 9 years did not significantly correlate with self-reported affect during any of the five conditions of the ERT ((*r_Spearman’s_rho_* <.|0.20|, *p* > 0.09). a. In the exploration of load and difference between ‘downregulating vs attending negative images’ and upregulating vs attending positive images’, no correlations with depressive load were observed (*r_Spearman’s_rho_* <.|0.21|, *p* > 0.072).

#### 3.2.3 Affect responsivity generally associated with MDD

Over all conditions, participants with an MDD diagnosis (within the previous nine years and/or currently) scored lower on self-reported affect (*M =* 51.97*, SD =* 18.99) than NDC participants (*M =* 56.97*, SD =* 19.65, *p =* 0.012). When tested per condition, MDD reported lower affect after upregulation of positive images (*M =* 66.61, *SD* = 13.58) than NDC (*M =* 72.38, *SD =* 66.71, *p =* 0.034, two-tailed, which did not survive correction for multiple correction). For the other conditions, no significant differences were found (see Table 1). The alpha level of 0.05 was SISA-Bonferroni corrected for the five tests (one per condition), considering the average correlation of 0.399 between the five affective ratings, leading to an alpha of 0.019.

**Table 1:** Mood responsivity in the MDD and NDC group.

	*M (SD)*		*U*	*p*
	MDD	NDC		
Attend neutral	56.10 (8.58)	59.68 (10.10)	1049.00	0.096
Attend negative	36.30 (13.57)	41.64 (17.26)	1098.50	0.19
Attend positive	64.53 (12.46)	68.75 (11.71)	1055.00	0.10
Downregulate negative	40.27 (12.98)	45.28 (15.11)	1054.00	0.11
Upregulate positive	66.61 (13.64)	72.38 (11.63)	983.50	0.034

*Note.* Overview of mood ratings per emotion regulation task condition in the MDD and NDC group, and test statistics for the comparison between these two groups. Mood ratings scale from 0 to 100 (“very sad” to “very happy”).

* *α <* 0.05, SISA-Bonferroni corrected for 5 tests.

Explorations of the difference scores between regulate and attend for the negative and positive conditions revealed no significant effect (‘downregulating vs attending negative images’: U = 1299.50, *p* = 0.95; ‘upregulating vs attending positive images’: U = 1146.50, *p* = 0.30). Within the MDD group, recency of MDD was not related to affective ratings (F(2,74) < 0.78, *p*s > 0.46).

### 3.3 Imaging results

Across participants, the task elicited brain regions commonly implicated in cognitive reappraisal (see Supplement 5).

#### 3.3.1 Brain functioning and disease load in MDD

Within the MDD group, depressive load was not significantly related to peak activity differences for the contrasts ‘downregulating vs attending negative images’ or ‘upregulating vs attending positive images’. Adding W6 clinical state characteristics (i.e., current depression diagnosis, IDS- and BAI scores, and medication use) at W6 as covariates did not change the results. Also, depression severity (IDS scores) was not associated with peak activity differences for the two contrasts.

**Fig. 2:**
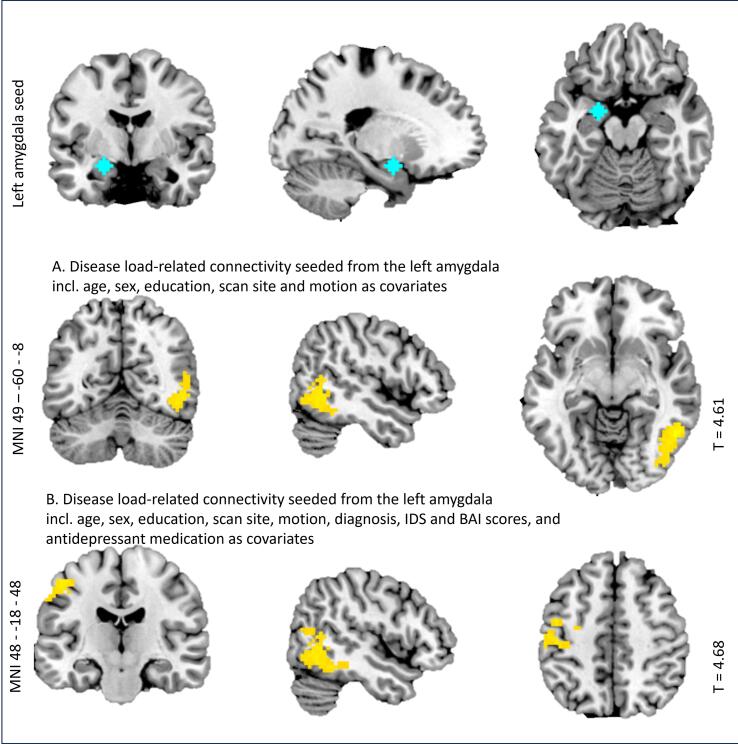

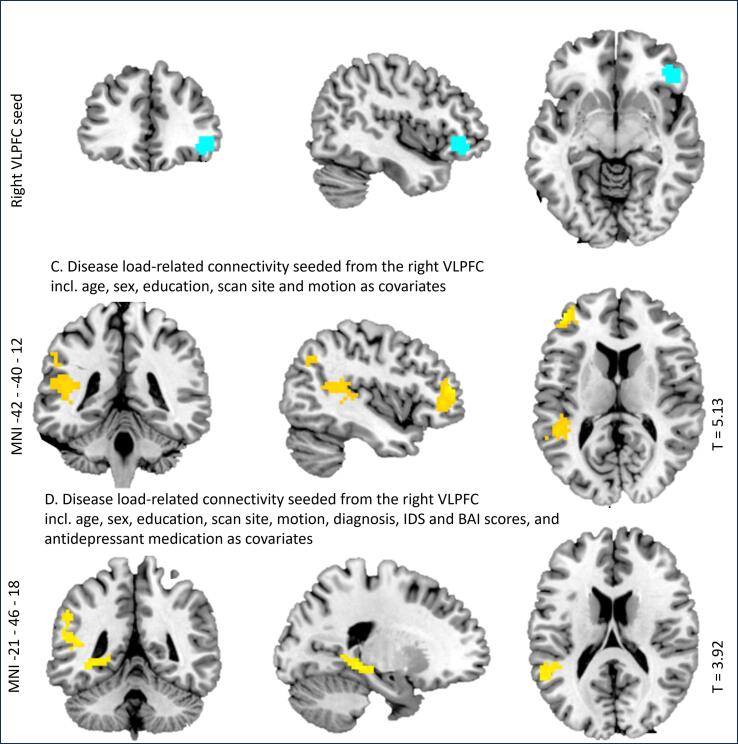
*Relation between disease load and functional connectivity during downregulation of negative emotions. Note.* Plots of significant relations with disease load and functional connectivity during downregulation (vs attending) of negative emotions, between the depicted clusters (in yellow) and the following seeds (in blue): 2A left amygdala seed; 2B left amygdala seed, including clinical covariates; 2C right VLPFC seed; 2D right VLPFC seed, including clinical covariates.

For the contrast ‘downregulating vs attending negative images’, disease load was positively related to functional connectivity between the left amygdala-seed and a cluster encompassing the inferior and middle temporal gyrus (Fig. 2A), and the right VLPFC-seed and clusters containing the left inferior and middle frontal gyrus and the left middle and superior temporal cortex, extending to the inferior parietal lobule (Fig. 2C). See Supplement 7 for the results table. After adding clinical state characteristics at W6 (MDD diagnosis, IDS- and BAI scores, and medication use at W6), the reported clusters remained and additional clusters were observed. Disease load additionally related positively to connectivity between (1) the left amygdala and a cluster including the post-central gyrus (see Fig. 2B) and (2) the right VLPFC seed and a cluster including the left parahippocampal gyrus and the hippocampus (see Fig. 2D; see Supplement 8 for the results table).

**Fig. 3:**
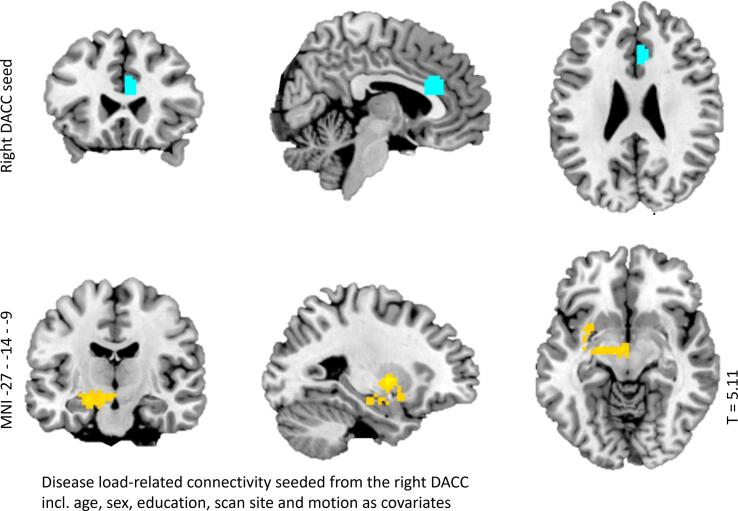
*Relation between disease load and functional connectivity during upregulation of positive emotions. Note.* Plots of significant relations with disease load and functional connectivity during upregulation (vs attending) of positive emotions, between the depicted cluster (in yellow) and the right DACC seed (in blue).

For the contrast ‘upregulating vs attending positive images’, disease load was negatively related to connectivity between the right dACC and a cluster including the putamen, amygdala, and the brain stem (substantia nigra) (see Fig. 3). Adding clinical state characteristics at W6 (MDD diagnosis, IDS- and BAI scores, and medication use at W6) as covariates of no interest changed the results in such a way that no cluster survived multiple comparison correction.

#### 3.3.2 Brain functioning generally associated with MDD

**Fig. 4:**
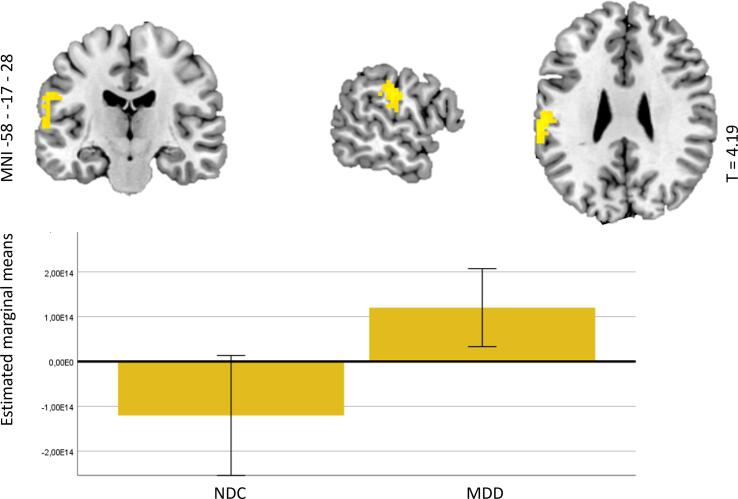
*BOLD responses during emotion regulation in MDD* vs *NDC. Note.* Clusters in which NDC showed significantly higher BOLD activity than MDD for the contrast ‘downregulating vs attending negative images’, together with plots of the estimated marginal means (plus 95% confidence intervals) of an ANCOVA on the values extracted from the contrast images in these clusters (including age, sex, education, scanner site and motion as covariates).

The MDD group showed lower peak activity in the right parietal cortex (encompassing the superior parietal gyrus and supramarginal gyrus) (see Fig. 4) than NDC during downregulation (vs attending) of negative emotions. No effects of group (MDD vs. NDC) were observed during upregulation (vs attending) of positive emotions.

**Fig. 5:**
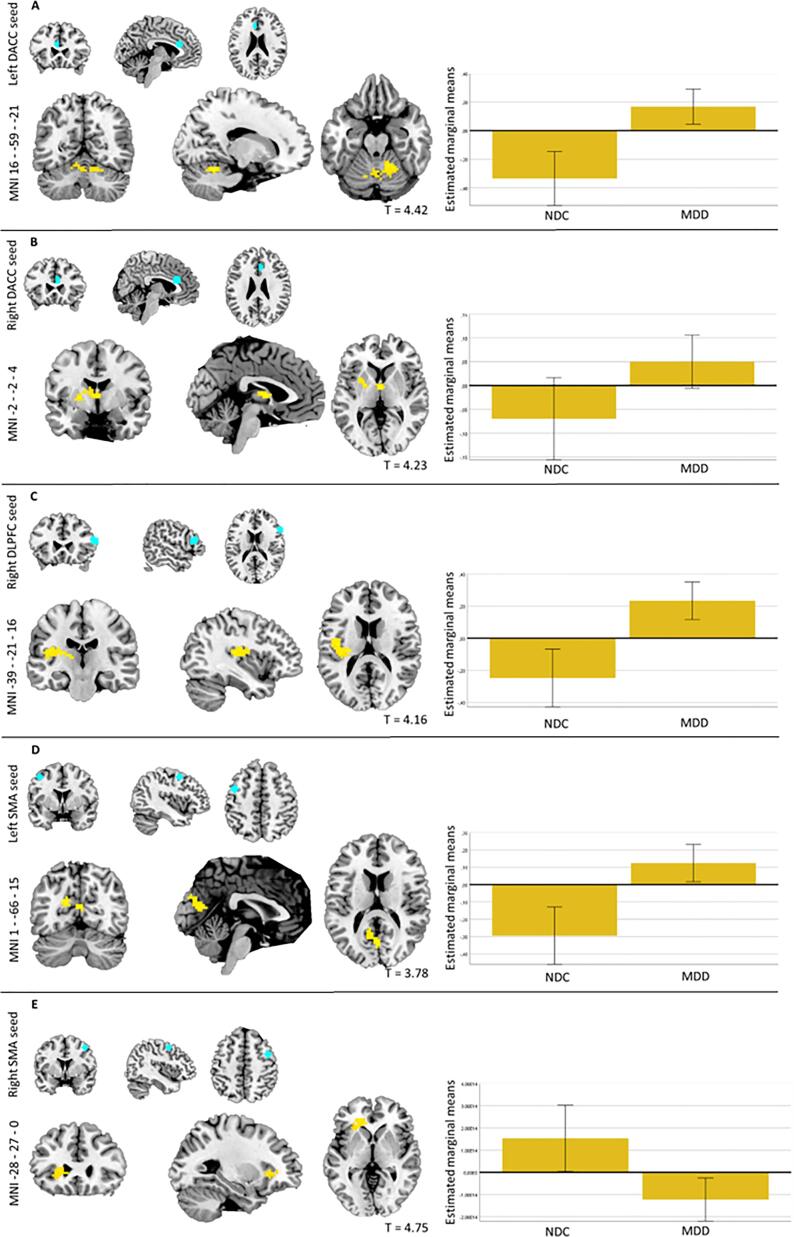
*Functional connectivity during emotion regulation in MDD* vs *NDC. Note.* Clusters in which MDD showed significantly higher functional connectivity compared with NDC for the contrast ‘upregulating vs attending positive images’, with plots of the estimated marginal means (plus 95% confidence intervals) based on an ANCOVA on the values extracted from contrast images in these clusters (including age, sex, education, scanner site and motion as covariates). 5A: left dACC seeded connectivity; 4B: right DACC seeded connectivity; 5C: right DLPFC seeded connectivity; 5D: left SMA seeded connectivity; 5E: right SMA seeded connectivity, all NDC vs MDD. The seeds are depicted in blue.

There were no significant differences in functional connectivity for the contrast ‘downregulating vs attending negative images’ between individuals with MDD and NDC. However, for the contrast ‘upregulating vs attending positive images’, individuals with MDD showed lower functional connectivity between (1) the left dACC-seed and the cerebellum (see Fig. 5A), (2) the right dACC-seed and the left anterior thalamus, putamen and insula (see Fig. 5B), and (3) the right DLPFC-seed and the left posterior insula / posterior thalamus (see Fig. 5C), (4) the left SMA-seed and a cluster encompassing the cuneus, precuneus, and posterior cingulate gyrus (see Fig. 5D), and (5) the right SMA-seed and a cluster involving the frontal operculum extending to the anterior insula (see Fig. 5E). See Supplement 9 for the results table and Supplement 10 for explorations of state dependency of effects, that suggested most effects to be independent of state.

## 4 Discussion

### 4.1 Summary

Given the high rates of recurrence and chronicity in people with MDD, a better understanding of mechanisms contributing to an unfavourable course is of major clinical importance. A history of depression constitutes a strong predictor of future episodes (Buckman et al., 2018), suggesting previous depression may increase vulnerability to a chronic or recurrent course. Here we aimed to examine the relation between neurocognitive mechanisms, specifically the neural correlates of emotion regulation, and disease load. We investigated the relation between regional brain activity during regulation of both negative and positive emotions and disease load, carefully characterized over the previous nine years. Next, we explored the relation of depressive load and functional connectivity from seeds commonly associated with emotion regulation, to investigate coordinated activity from these regions in relation to cumulative load. Furthermore, we explored whether these brain functioning abnormalities were related to disease load specifically, or a more general marker of MDD, by (1) controlling for clinical state characteristics and (2) exploring overlap with effects of general occurrence of depression by comparing individuals with an MDD diagnosis (currently and/or in the previous nine years) to NDC.

Disease load did not relate to reported changes in *affect* or to brain *activity* during the emotion regulation task. It did, however, relate to *connectivity* of brain regions when regulating emotions. Specifically, during the regulation of negative emotions, disease load was related to connectivity between the amygdala-seed and VLPFC-seed with regions typically implicated in integrating or regulating emotional information (including the inferior and middle temporal gyri, fusiform gyrus, inferior and middle frontal gyrus and inferior parietal cortex, and after correction for clinical variables also with the pre- and postcentral gyri and parahippocampal gyrus and hippocampus). These load-related alterations in functional connectivity did not overlap with disorder-general abnormalities. During the upregulation of positive emotions, disease load was associated with the connectivity between the right DACC-seed and the amygdala and putamen, an association which did not survive multiple comparison correction after controlling for current clinical characteristics, and which (partly) spatially overlapped with disorder-general abnormalities.

### 4.2 Neural emotion regulation capacity as a function of depressive disease load

Contrary to our expectations, disease load within individuals with MDD was not related to peak activity during emotion regulation. Also, no relation was found with negative or positive affect following the task conditions, suggesting that previous depression does not impact immediate affective outcome in the context of an emotion regulation task. Nevertheless, disease load may relate to impaired regulation in more compelling real life emotional or self-relevant situations. Alternatively, severity of current depression and not necessarily the cumulative load of previous depression may predominantly impact brain activity and affect reactivity. However, no results were found when we explored relations with current symptom severity directly.

Instead, how certain brain areas are functionally connected appeared relevant for understanding the effect of previous disease load. Our explorative findings of positive relations between disease load and connectivity between emotion regulation-related areas suggests that the coherence between recruited regions rather than the extent to which regions are recruited may be important for understanding the increased risk for depressive recurrence and chronicity. Interestingly, both the left amygdala and right VLPFC showed positive relations between load and connectivity with the lateral temporal gyri during downregulation of negative emotions. The temporal lobe can, in the context of an emotion regulation task, be viewed as an intermediate integration area between the limbic affective appraisal system and the frontoparietal control system (Ochsner et al., 2012). Our results might suggest higher disease load relates to heightened calling for neural resources supporting the integration of affective valuation of salient emotional stimuli within an individuals’ context and for regulatory control (Ochsner et al., 2012). Heightened connectivity between the left amygdala-seed (primary emotional processing area) and the temporal and occipital lobe and the postcentral and fusiform gyri (areas related to the integration of perceptual and interoceptive aspects of information (Ochsner et al., 2012) as a function of previous disease load during downregulating negative emotions suggests enhanced (sensorial and contextual) integration of negative emotional information within an emotion regulation network (Morawetz et al., 2017). Furthermore, a positive relation between disease load and connectivity between the right VLPFC and inferior and middle frontal and inferior parietal areas (all within a frontoparietal regulatory network) during downregulation of negative emotions, suggests disease load relates to the cognitive demand involved in both calling upon but also maintaining top-down cognitive reformulation of mental representations (Kohn et al., 2014, Frank et al., 2014, Buhle et al., 2014) as well. Furthermore, the relation between disease load and connectivity between the right VLPFC and the hippocampus and parahippocampus (which was found after clinical covariates were added) suggests higher (negative) emotional sensitivity and memory integration during regulatory control (Ochsner et al., 2012) with higher previous disease duration and/or burden.

Increased connectivity between these integration and regulation areas as a function of previous disease load may reflect increased need for regulatory alignment, which might fall short in the context of sustained daily life stress and negative life events. This may contribute to a shift towards increased perceptual, contextual and declarative memory integration of negative salient information, ultimately contributing to lower affect and depressed mood, making these connectivity patterns relevant to understanding long-term unfavourable course. The current findings suggest that these connectivity patterns vary as a function of cumulative depression and therefore possibly reflect a scarring effect of the cumulative duration and severity of previous depressive symptomatology. Given that the number of previous episodes has been found predictive of future recurrence (Kessing et al., 2004, Lewinsohn et al., 1989, Buckman et al., 2018), these connectivity patterns may mediate the risk for future persistence or recurrence of depressive symptomatology. It is unlikely that these load-related connectivity patterns during downregulation of negative emotions reflect a more general depression-related pathology because the results of the disease load- and MDD vs. NDC-analyses did not spatially overlap and controlling for current state characteristics did not alter most results. Additionally, when exploring relations with severity of current depressive symptomatology, no relations were observed. Alternatively, our results may represent pre-existent neural abnormalities, which put individuals at risk for enduring invalidating periods of depression. Nevertheless, these load-related connectivity patterns do not simply appear to be a magnification of general disease-related abnormalities, but may, together with disease-general vulnerability mechanisms, account for individual differences in vulnerability to persistent or recurrent course.

Regarding upregulation of positive emotions, we found a negative relation between disease load and connectivity between the right DACC and the amygdala and putamen. These areas have been implicated in emotional salience detection and detection for the need of regulatory control (Ochsner et al., 2012), suggesting disease load relates to lower processing and employment of regulation when instructed to upregulate positive emotions. However, these results did not hold when we controlled for current clinical state and antidepressant medication. Also, explorative plots showed that brain functioning in the putamen-cluster related to the DACC was related to recency of diagnosis at the time of scanning (see Supplement 10), suggesting (a trend towards) state dependency. Furthermore, connectivity between the right DACC and the putamen spatially overlapped with MDD-general abnormal connectivity between these regions during upregulating positive emotions. An effect of disease load on the connectivity between these limbic structures during upregulation of positive emotions can thus be seen as an aggravated function of general MDD-related pathology.

### 4.3 Neural emotion regulation abnormalities in general associated with MDD

Load-related associations during downregulating negative emotions did not overlap with MDD-related (i.e., fulfilling criteria for MDD at some point in the past nine years) associations (also not subthreshold), but MDD-related abnormalities were observed that could represent a general vulnerability to the occurrence of depressive symptomatology (with no specific relation to duration or course of these depressive symptoms). Where *connectivity* between areas relevant to successful downregulation of negative emotions was related to previous disease load, lower *activity* in the parietal cortex during downregulation of negative emotions seems characteristic for (current or previous) MDD in general. Visualizations of activity in these regions with respect to current versus short and long remission status indicated that the parietal hypoactivity was observed independent of depressed state (see Supplement 10). Involvement of the superior parietal cortex is generally considered important for inhibitory control over negative emotional responses, with superior parietal activity being especially relevant for reappraisal or distancing (Frank et al., 2014, Buhle et al., 2014, Ochsner et al., 2004, Picó-Pérez et al., 2018). Activity within this area has previously been found lacking in depressed individuals (Zilverstand et al., 2017, Picó-Pérez et al., 2017, Sheline et al., 2009, Rive et al., 2013). Even though these regulatory brain areas were less activated, individuals in the MDD group did not show significantly lower affect upon task conditions involving downregulation of negative emotional pictures than NDC, suggesting (near) normal regulation success on a behavioural level. Nevertheless, neural regulatory capacity may fall short in the face of enduring negative emotions and stress in individuals vulnerable to experiencing MDD.

During upregulation of positive emotions, no *activity* abnormalities were observed between MDD and controls. Instead, *connectivity* between brain areas involved in regulatory control and integrating sensory, emotional, and cognitive information differed between these groups. These results did partly overlap with load-related associations. More specifically, increased connectivity during upregulation of positive emotions was observed between the DACC and the cerebellum, putamen, anterior insula and thalamus, of which the putamen cluster was spatially similar to the area in which connectivity was found with the DACC-seed in relation to disease load. Other connectivity abnormalities in MDD (vs NDC) were not similar to load-related findings. During upregulation of positive emotions, MDD showed increased connectivity vs NDC between the DLPFC and posterior insula and thalamus, and the SMA and the cuneus, precuneus, posterior cingulate cortex, insula and frontal operculum, with no effects of current depressive state (see Supplement 10). Given the involvement of these areas in salience processing and emotion regulation (Seeley, 2019, Uddin, 2015, Nomi et al., 2016), these MDD-characteristic connectivity patterns may reflect higher vigilance or discomfort when experiencing positive emotions and greater regulatory effort when instructed to increase positive emotions, which may underpin the difficulties in savouring positive emotions in daily life that are associated with depression (Liu and Thompson, 2017). Indeed, individuals with MDD showed a trend towards lower affective ratings after task conditions involving upregulation of positive emotions than NDC (although not significant after correction for multiple comparisons). Further research is needed to better comprehend brain functioning during regulation of positive emotions in MDD.

Most of these abnormalities were found irrespective of current depressed state, suggesting that abnormalities in networks related to emotional processing and regulation generally relate to a (past) presence of depression. Since they seem related to general occurrence of depression, they may represent a trait-like vulnerability to the manifestation of depression. However, whether they also reflect a vulnerability to the development of a first episode cannot not be derived from the current data.

### 4.4 Strengths and limitations

One of the strengths of the current study is the prospective design of the NESDA study, enabling a detailed prognostic assessment of disease load over a long period of time (nine years). Previous studies have either studied disease severity and duration of symptoms separately, but using unique longitudinal data on symptom course, we were able to create a measure which captures both aspects, and thereby may better represent the burden of MDD. Furthermore, the present study provides a comprehensive and extensive examination of the neurobiological correlates of emotion regulation in relation to MDD course, assessing both negative and positive emotionality and both peak activity and connectivity.

However, limitations should be mentioned as well. Since the neuroimaging data are cross-sectional, causal inferences could not be made. Also, any conclusions that can be drawn from this dataset are limited to the regulation of visually evoked emotional responses, given the use of images in our task design. Furthermore, each Emotion Regulation Task condition was only repeated three times, which may have led to an underpowered dataset. However, each block consisted of four images, shown for eight seconds, leading to 96 s per condition. This seems sufficient to calculate reliable task effects (see Supplement 5 for the main task effects). Another limitation of this study is the possible effect of antidepressant medication use on the present findings, even though we corrected for current medication use within the disease load analyses. Also, participants were scanned on different sites, which may enlarge between-subject variability. However, similar scanning protocols were used to limit between-site variability, and scanner site was added as two dummy variables in all analyses. Furthermore, even though the combined duration-severity measure of disease load has several benefits, it provides limited insight in different symptom profiles. Supplement 11 provides plots of the relation with functional connectivity results, split out for duration and severity. These plots suggest the results are driven more by the severity of symptoms than by the duration of symptoms. Also, the selection of seeds could have been differently, possibly leading to different results. The current selection was, however, based on extensive previous research and therefore informs on the connectivity of brain regions involved in brain networks previously associated with MDD and emotion regulation. As a final note, the results in the current study should be considered explorative, serving as a basis for hypothesis-driven testing in future work, and not as definite findings.

### 4.5 Conclusions

In conclusion, functional connectivity during regulation of emotions seems relevant for understanding the effects of previous duration and severity of depressive symptomatology (disease load). Connectivity between areas implicated in both processing and regulating negative emotions was related to previous disease load. These connectivity patterns may reflect and potentially predispose to overly processing of negative emotional information, but also impaired regulatory control and insufficient use of self-related thoughts for employment in cognitive reappraisals. Altogether, this may not only facilitate but also maintain negative affective states in the long run, facilitating recurrent or chronic disease course. These abnormalities were not simply a magnification of general depression-related pathology or a reflection of current state, since they did not spatially overlap with abnormalities in general related to MDD and were not affected by correction for clinical state characteristics. The relation between disease load and connectivity between the DACC and the amygdala and putamen during upregulation of positive emotions may have been driven by current symptomatic state.

In sum, these findings suggest that load-related functional connectivity patterns during regulation of emotions do not simply represent an aggravation of general psychopathological variance. Instead, they may represent a built-up vulnerability to unfavourable course, on top of disease-general emotion regulation abnormalities. These insights may provide guidance to improvement of therapeutic interventions. Further longitudinal research is necessary to confirm the causal relation between disease load and alterations in neural processing in frontoparietal and cingulo-opercular networks, and its relevance to understanding unfavourable course of depression.

**Funding and Disclosure**.

The infrastructure for the NESDA study (https://www.nesda.nl) is funded through the Geestkracht program of the Netherlands Organization for 10.13039/100005622Health Research and Development (Zon- Mw, grant no. 10-000-1002). This study is supported by the participating universities and mental health-care organizations: VU University Medical Center Amsterdam, University Medical Center Groningen, Leiden University Medical Center, GGZ inGeest, Arkin, GGZ Rivierduinen, Lentis, GGZ Friesland, GGZ Drenthe, Scientific Institute for Quality of Healthcare (IQ healthcare), Netherlands Institute for Health Services Research (NIVEL) and Netherlands Institute of Mental Health and Addiction (Trimbos Institute).

The authors have nothing to disclose.

## Declaration of Competing Interest

The authors declare that they have no known competing financial interests or personal relationships that could have appeared to influence the work reported in this paper.

## Data Availability

Data will be made available on request.
