# Supplementary material for: Functional MRI correlates of emotion regulation in major depressive disorder related to depressive disease load measured over nine years
Source: Neuroimage Clin. 2023 Nov 11;40:103535. doi: 10.1016/j.nicl.2023.103535 (PMC10696117; doi:10.1016/j.nicl.2023.103535)
Supplement: Supplementary data 1 [file mmc1.pdf]

Supplementary materials to *Functional MRI correlates of emotion regulation in Major Depressive Disorder related to depressive disease load measured over nine years*

Supplement 1 IAPS pictures used in the Emotion Regulation Task

Pictures in the negative category contained both sad and fearful images. Within each category, pairs were created with pictures containing similar content, so pictures for attend- and regulate conditions could easily be matched. The pairs for the two conditions were matched on activity, complexity and type of scene, but also on valence, arousal and dominance (see Table S1). Ratings for valence, arousal and dominance were derived from Mikel et al. (2005) and Lang et al. (1999). Fearful and sad pictures match on valence, but not arousal. Sad and positive pictures match on arousal, but not on valence. Only pictures with relatively low standard deviation were chosen, to avoid ambiguity.

Negative (sad) pictures selected from the IAPS database (in pairs): 3220 & 3230; 3301 & 3300; 2900 & 2800; 3350 & 2053; 2205 & 2141; 9040 & 9075.
Negative (fearful) pictures selected from the IAPS database (in pairs): 6520 & 3530; 6231 & 6260; 9050 & 9600; 6350 & 6510; 9921 & 9940; 9410 & 9163.
Positive pictures selected from the IAPS database (in pairs): 2209 & 2300; 8080 & 8210; 2216 & 2306; 5621 & 8370; 8497 & 2389; 4599 & 4623; 8200 & 8190; 1710 & 1463; 2314 & 2035; 8540 & 8120; 2340 & 2598; 2395 & 2370.
Neutral pictures selected from the IAPS database (in pairs): 2850 & 2570; 7493 & 2235; 2393 & 2191; 2397 & 2396; 2411 & 2383; 2377 & 2374; 2191 & 2200.

		M(SD)		
Category	Pair	Valence	Arousal	Dominance
Negative				
Sad	Pairs 1	2.04 (1.39)	5.32 (2.15)	3.43 (2.01)
	Pairs 2	2.19 (1.44)	5.29 (2.21)	3.55 (2.12)
Fearful	Pairs 1	2.05 (1.39)	6.78 (2.01)	2.95 (2.08)
	Pairs 2	2.10 (1.39)	6.79 (2.07)	2.84 (2.07)
Positive	Pairs 1	7.5 (1.38)	5.25 (2.11)	6.13 (1.87)
	Pairs 2	7.54 (1.40)	5.18 (2.18)	6.15 (2.01)
Neutral	Pairs 1	5.30 (1.23)	3.20 (1.97)	0.79 (3.29)
	Pairs 2	5.23 (1.30)	3.32 (1.97)	0.82 (3.29)

Supplementary Table 1. Scores on valence, arousal, dominance and luminance of pictures selected from the IAPS for the ERT.

Supplement 2 SPM12 fMRI pre-processing scripts

Realignment

```
matlabbatch{1}.spm.spatial.realign.estwrite.data = {coregistered functional scans};
matlabbatch{1}.spm.spatial.realign.estwrite.eoptions.quality = 0.9;
matlabbatch{1}.spm.spatial.realign.estwrite.eoptions.sep = 4;
matlabbatch{1}.spm.spatial.realign.estwrite.eoptions.fwhm = 5;
matlabbatch{1}.spm.spatial.realign.estwrite.eoptions.rtm = 1;
matlabbatch{1}.spm.spatial.realign.estwrite.eoptions.interp = 4;
matlabbatch{1}.spm.spatial.realign.estwrite.eoptions.wrap = [0 0 0];
matlabbatch{1}.spm.spatial.realign.estwrite.eoptions.weight = '';
matlabbatch{1}.spm.spatial.realign.estwrite.roptions.which = [2 1];
matlabbatch{1}.spm.spatial.realign.estwrite.roptions.interp = 4;
matlabbatch{1}.spm.spatial.realign.estwrite.roptions.wrap = [0 0 0];
matlabbatch{1}.spm.spatial.realign.estwrite.roptions.mask = 1;
matlabbatch{1}.spm.spatial.realign.estwrite.roptions.prefix = 'r';
```

Coregistration

```
matlabbatch{1}.spm.spatial.coreg.estimate.ref = {functional scans};
matlabbatch{1}.spm.spatial.coreg.estimate.source = {anatomical scan};
matlabbatch{1}.spm.spatial.coreg.estimate.other = {''};
matlabbatch{1}.spm.spatial.coreg.estimate.eoptions.cost_fun = 'nmi';
matlabbatch{1}.spm.spatial.coreg.estimate.eoptions.sep = [4 2];
matlabbatch{1}.spm.spatial.coreg.estimate.eoptions.tol = [0.02 0.02 0.02 0.001 0.001 0.001 0.01 0.01
0.01 0.001 0.001 0.001];
matlabbatch{1}.spm.spatial.coreg.estimate.eoptions.fwhm = [7 7];
```

Normalization

```
matlabbatch{1}.spm.spatial.normalise.estwrite.subj.vol = {anatomical scan};
matlabbatch{1}.spm.spatial.normalise.estwrite.subj.resample = {functional scans};
matlabbatch{1}.spm.spatial.normalise.estwrite.eoptions.biasreg = 0.0001;
matlabbatch{1}.spm.spatial.normalise.estwrite.eoptions.biasfwhm = 60;
matlabbatch{1}.spm.spatial.normalise.estwrite.eoptions.tpm =
{'/data/cnc/software/spm/spm12/v7487/v7487/tpm/TPM.nii'};
matlabbatch{1}.spm.spatial.normalise.estwrite.eoptions.affreg = 'mni';
matlabbatch{1}.spm.spatial.normalise.estwrite.eoptions.reg = [0 0.001 0.5 0.05 0.2];
matlabbatch{1}.spm.spatial.normalise.estwrite.eoptions.fwhm = 0;
matlabbatch{1}.spm.spatial.normalise.estwrite.eoptions.samp = 3;
```

matlabbatch{1}.spm.spatial.normalise.estwrite.woptions.bb = [-78 -112 -70
78 76 85];
matlabbatch{1}.spm.spatial.normalise.estwrite.woptions.vox = [3 3 3];

Smoothing

matlabbatch{1}.spm.spatial.smooth.data = {functional scans};
matlabbatch{1}.spm.spatial.smooth.fwhm = [8 8 8];
matlabbatch{1}.spm.spatial.smooth.dtype = 0;
matlabbatch{1}.spm.spatial.smooth.im = 0;
matlabbatch{1}.spm.spatial.smooth.prefix = 's';
matlabbatch{1}.spm.spatial.normalise.estwrite.woptions.interp = 7;
matlabbatch{1}.spm.spatial.normalise.estwrite.woptions.prefix = 'w';

Supplement 3 GPPI seed information

Seed regions were based on main task effects, in combination with literature on areas commonly involved in emotional processing and regulation (Buhle et al., 2014; Frank et al., 2014; Kohn et al., 2014), and involved the left and right amygdala, dorsal anterior cingulate cortex, dorsolateral PFC, ventrolateral PFC, supplementary motor area and superior parietal cortex. We created 6 mm spheres around peak coordinates from main task effects or around significant voxels more similar to the coordinates reported in the literature.

		x	y	z
Amygdala	Left	-21	-4	-16
	Right	24	-4	-19
DACC	Left	-3	26	22
	Right	3	26	22
DLPFC	Left	-57	23	14
	Right	57	23	14
Lateral parietal	Left	-54	-49	29
	Right	54	-49	29
SMA	Left	-42	2	50
	Right	42	2	50
VLPFC	Left	-45	32	-7
	Right	45	32	-7

Supplementary Table 2. Seed coordinates for gPPI analyses

Supplement 4 Demographic and clinical characteristics

MDD sample

Demographic and clinical characteristics for the MDD sample can be found in Supplementary Table 3.

NDC sample

Demographic and clinical characteristics for the NDC group can be found in Supplementary Table 3.

MDD vs NDC

Education level did not differ between the MDD and the NDC sample, however the depressed sample was older (p=.032) and consisted of a higher proportion of women than the NDC sample (p=.003). Furthermore, MDD patients showed significantly higher IDS scores (p<.001) and BAI scores (p<.001) at time of scanning than NDC.

	MDD	NDC	U / X^2	*p*
N	77	35		
Age	47.16 (10.14)	52.31 (8.76)	1041.00	.032
Sex ratio	25 M / 62 F	19 M / 16 F	7.07	.008
Education level – *M* (*SD*)	5.98 (1.81)	6.49 (1.95)	1064.00	.052
IDS score at W6 – *M* (*SD*)	19.16 (9.85)	3.56 (2.52)	130.50	<.001*
BAI score at W6 – *M* (*SD*)	8.97 (6.58)	2.44 (2.92)	415.50	<.001*
Disease load over W1-W6 – *M* (*SD*)	0.46 (0.14)			
Months with symptoms over W1-W6 – *M (SD)*	52.51 (30.50)			
Comorbid anxiety disorders at W6				
Generalized anxiety disorder	6	0		
Panic disorder	10	0		
Social anxiety disorder	14	0		
Comorbid anxiety disorders lifetime				
Generalized anxiety disorder	52	0		
Panic disorder	66	0		
Social anxiety disorder	44	0		

Supplementary Table 3. Demographic and clinical characteristics of MDD patients and NDC.

Diagnosis-subgroups within the MDD sample

For interpretation reasons, we explored characteristics in different recency subgroups within our MDD sample. Current depression was defined as a CIDI-derived MDD diagnosis within the last month prior to scanning. Recent remission was defined as a CIDI-derived MDD diagnosis between six months and one month prior to scanning. Long-term remission was defined as a CIDI-derived MDD diagnosis between nine years and six months prior to scanning. See Supplementary Table 4 for demographic and clinical information split out per recency subgroup.

	Current depression	Recent remission	Long-term remission
N	28	20	29
Age	51.29 (8.64)	45.85 (7.69)	44.07 (11.79)
Sex ratio	9 M / 19 F	9 M / 11 F	7 M / 22 F
Education level – *M* (*SD*)	6.21 (1.69)	5.85 (1.98)	5.86 (1.87)
IDS score at W6 – *M* (*SD*)	23.28 (9.01)	20.35 (10.25)	14.38 (8.46)
BAI score at W6 – *M* (*SD*)	11.04 (6.51)	9.10 (5.25)	6.90 (7.01)
Disease load over W1-W6 – *M* (*SD*)	0.48 (0.13)	0.45 (0.15)	0.45 (0.15)

Supplementary Table 4. Demographic and clinical characteristics of subgroups within the MDD sample

Distribution of disease load within the MDD sample

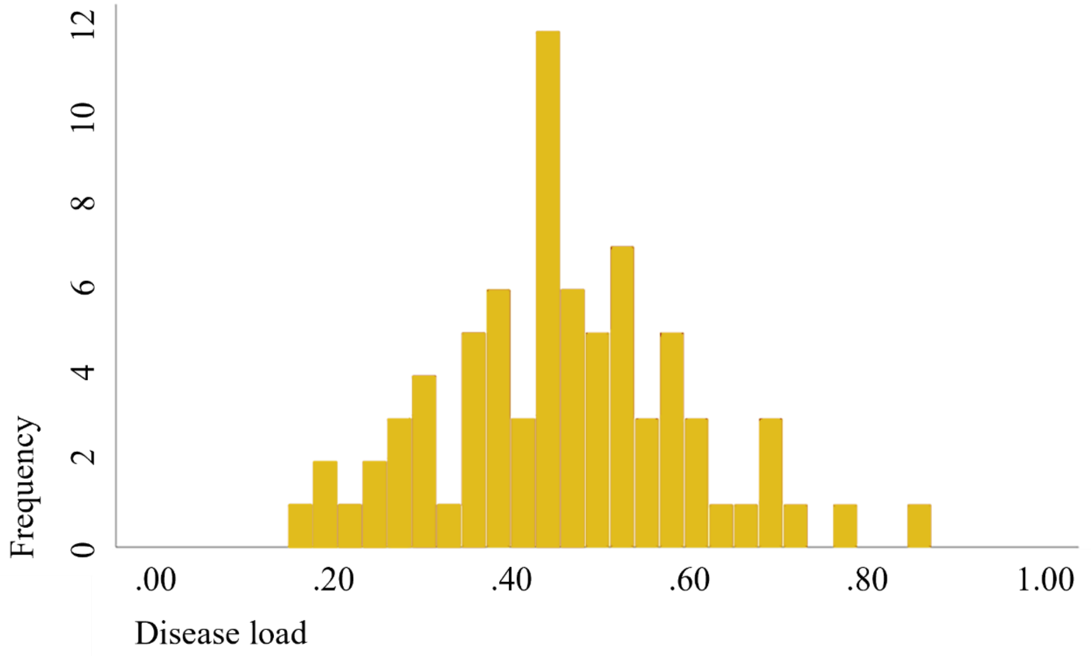

Supplementary Figure 1. Distribution of the disease load over W1 to W6 in the MDD group.

Supplement 5 Main task effects

To ensure the Emotion Regulation Task (ERT) was performing properly, we tested the effect of task over all participants, for the contrast image regulate vs attend (negative and positive combined). Cluster-based inference was based on a significance level of $p<0.05$, family-wise error rate corrected for multiple comparisons, based on non-parametric permutation testing (5000 permutations). The task elicited regions commonly implicated in cognitive reappraisal, see Supplementary Figure 2.

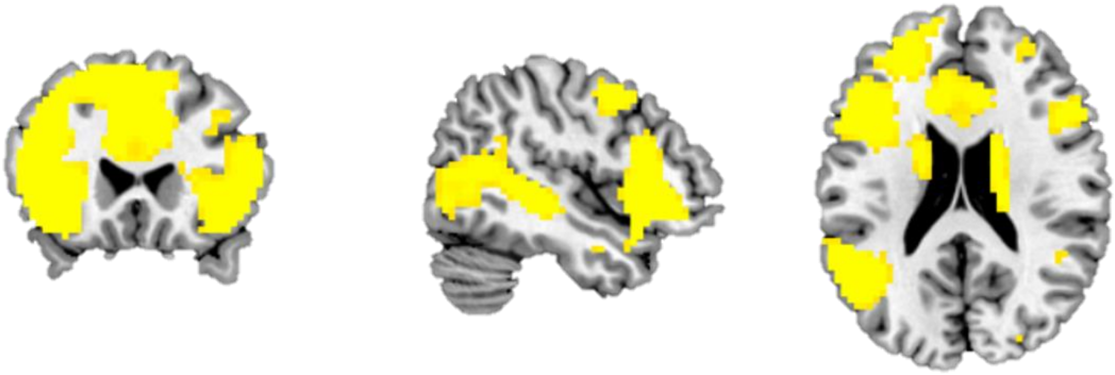

Supplementary Figure 2. Main task effects regulate > attend across positive and negative valenced pictures.

Supplement 6 Relation between affect responsivity and disease load

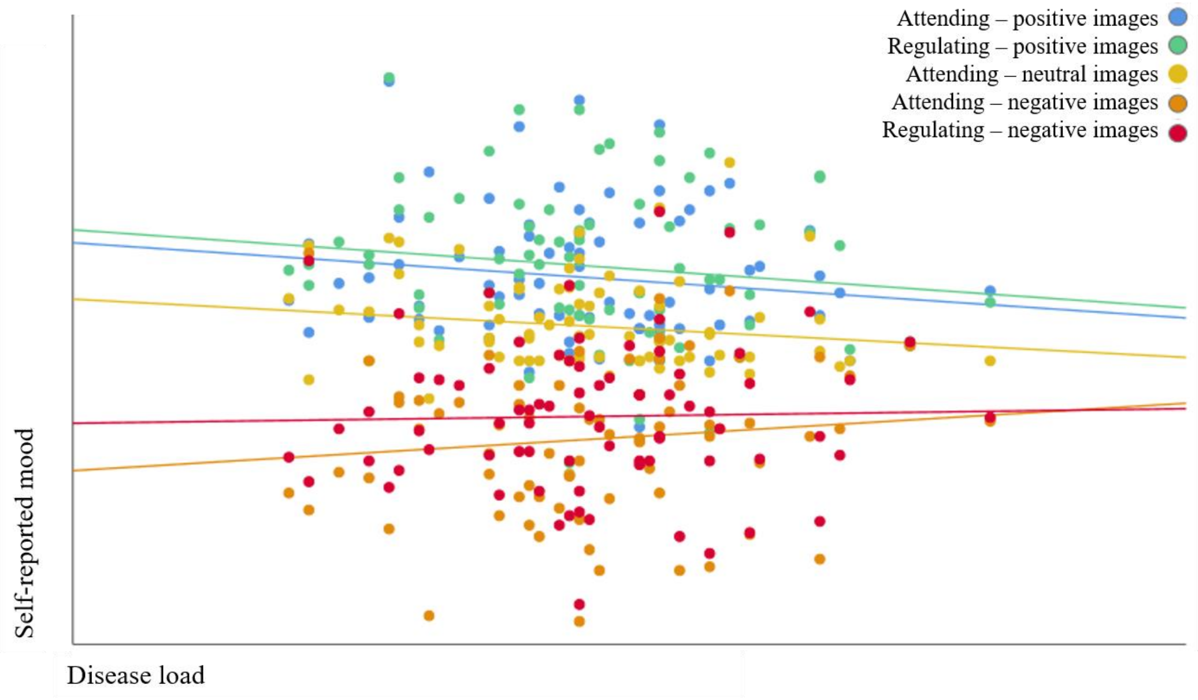

Supplementary Figure 3. Relation between disease load and mood-responsivity: lines of best fit showing non-significant relationships between disease load over W1-W6 and self-reported mood during the Emotion Regulation Task in MDD patients, split out per task condition.

		Disease load
Attending - neutral	Correlation Coefficient	-0.190
	Sig. (2-tailed)	.098
Attending - negative	Correlation Coefficient	0.110
	Sig. (2-tailed)	.342
Attending - positive	Correlation Coefficient	-0.117
	Sig. (2-tailed)	.311
Regulating - negative	Correlation Coefficient	0.025
	Sig. (2-tailed)	.830
Regulating - positive	Correlation Coefficient	-0.121
	Sig. (2-tailed)	.296
	Correlation Coefficient	-.194
	Sig. (2-tailed)	.091

Difference regulating – attending negative images	Correlation Coefficient	-0.85
Difference regulating – attending positive images	Sig. (2-tailed)	.464

Supplementary Table 5. Spearman’s rho correlations with two-tailed significance.

Supplement 7 Table for brain connectivity in relation to depressive load

Seed	Side	Area	*p*-value	k	T	MNI-coordinates x	y	z
Downregulating vs attending negative images								
Amygdala left	R	Inferior temporal gyrus / Middle temporal gyrus / Occipital gyrus / Fusiform gyrus	0.014	222	4.61	51	-55 -	-4
					4.11	42	67	-10
					3.94	51	-58	5
VLPFC right	L	Inferior frontal gyrus/Middle frontal gyrus	0.049	107	5.13	-42	50	11
					4.26	-39	41	-1
					3.89	-45	41	17
	L	Superior temporal gyrus / Inferior parietal lobe	0.011	323	4.19	-57	-46	17
					4.13	-45	-46	14
					4.02	-57	-49	32
Upregulating vs attending positive images								
DACC right	L	Putamen / Amygdala	0.024	174	5.11	-27	-4	2
					4.19	-21	-13	-13
					3.93	-30	2	-10

Table 6. Results of functional connectivity analyses in the MDD sample for the relation with disease load, for the contrast ‘downregulating vs attending negative images’. *P*-values are FWE corrected for multiple comparisons, with age, sex, education and scan site (2 dummies) added as covariates.

Supplement 8 Brain connectivity in relation with disease load with clinical characteristics included as covariates

We additionally ran the statistical analyses on disease load with MDD diagnosis at W6 (yes/no), IDS and BAI scores at W6, and antidepressant medication use (dummy) at W6 in one model to correct for possible effects of clinical state. For the BOLD response peak activation analyses, we again found no relation with disease load. For the functional connectivity analyses, we found similar results during negative downregulation vs negative attending (see Supplementary Table 3).

Seed	Side	Area	*p* (FWE-corrected)	k	T	x	y	z
Downregulating negative images								
Amygdala left	R	Inferior temporal gyrus / Middle temporal gyrus / Occipital gyrus / Fusiform gyrus	0.008	286	4.68	54	-55	-4
					4.45	42	-67	-10
					3.97	51	-58	5
	L	Postcentral gyrus	0.023	154	4.4	-42	-22	44
					4.24	-42	-22	53
					4.19	-48	-16	53
VLPFC right	L	Parahippocampus / Hippocampus	0.034	140	3.92	-21	-37	-4
					3.89	-33	-31	-7
					3.82	-36	-46	-4
	L	Superior temporal gyrus / Inferior parietal lobe	0.048	106	3.81	-57	-46	17
					3.79	-45	-46	14
					3.64	-54	-40	35

Table 7. Results of group differences in fMRI functional connectivity in the MDD sample for the relation with disease load, for the contrast negative regulation vs negative attend, including clinical characteristics at W6 as covariates of no interest.

Supplement 9 Tables for brain functioning in MDD patients compared to NDC

	Side	Area	*p*-value	k	T	MNI-coordinates x	y	z
NDC vs MDD (whole brain)	L	Superior parietal cortex	0.034	114	4.19	-63	-25	29
					3.48	-63	-13	8
					3.36	-60	-43	32

Supplementary Table 8. Results of group differences in fMRI BOLD responses in the NDC vs MDD sample, for the contrast ‘downregulating vs attending negative images’. *p*-values are FWE-corrected for multiple comparisons at the cluster level, controlled for age, sex, education, scan site (2 dummy variables), and motion (mean Framewise Displacement).

Seed	Side	Area	*p*	k	T	MNI-coordinates x	y	z
DACC left	R	Cerebellum	0.035	107	4.42	15	-49	-19
					3.72	3	-64	-19
					3.6	-9	-61	-16
DACC right	L	Thalamus (ventral anterior	.0040	106	4.23	0	-4	8
	L	Putamen			3.97	-21	-1	5
		Insula			3.93	-27	5	2
DLPFC right	L	Posterior insula/Thalamus	0.020	153	4.15	-42	-13	20
					4.03	-33	-25	14
					3.85	-42	-22	17
SMA left	L	Cuneus	0.019	136	3.87	3	-70	14
		Precuneus			3.86	-15	-67	20
		Cingulate gyrus				-6	-58	14
SMA right	L	Frontal operculum/ Anterior Insula	0.042	105	4.75	-18	35	2
					4.3	-24	23	2
					4.03	-9	32	-1

Supplementary Table 9. Results of differences in fMRI functional connectivity between NDC and MDD (all NDC < MDD), for the contrast ‘upregulating vs attending positive images’. *P*-values are FWE corrected for multiple comparisons at the cluster level, with age, education, sex and scan site (two dummy variables), and motion (mean Framewise Displacement) added as covariates.

Supplement 10 Plots of estimated marginal means within clusters significantly related to disease load or MDD in general, per diagnostic subgroup

Disease load

To further explore the relation with clinical state of disease-load related findings, we additionally plotted the estimated marginal means of an ANCOVA on the contrast-values extracted from the significant clusters, split out for subgroups of patients (current depression, recent remission [1-6 month remission], long-term remission [>6 months]).

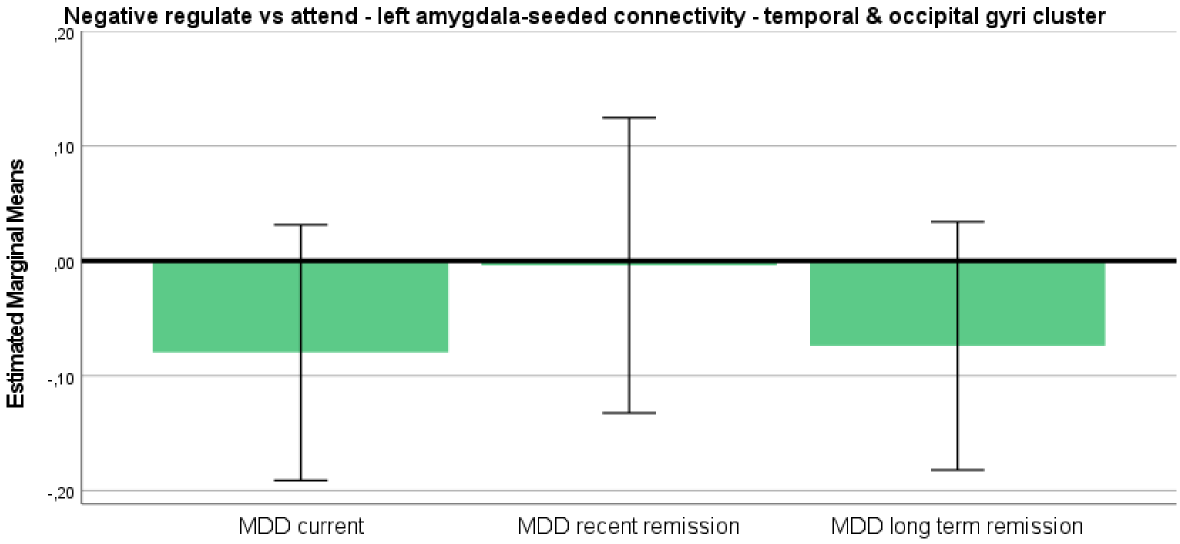

Supplementary Figure 4. Temporal and occipital gyri clusters with which connectivity with the left amygdala-seed was related to previous disease load during downregulation of negative emotions, including a plot of the estimated marginal means (plus 95% confidence intervals) of an ANCOVA on the contrast images within this cluster (including age, sex, education, scanner site and motion as covariates), split out per MDD subgroup.

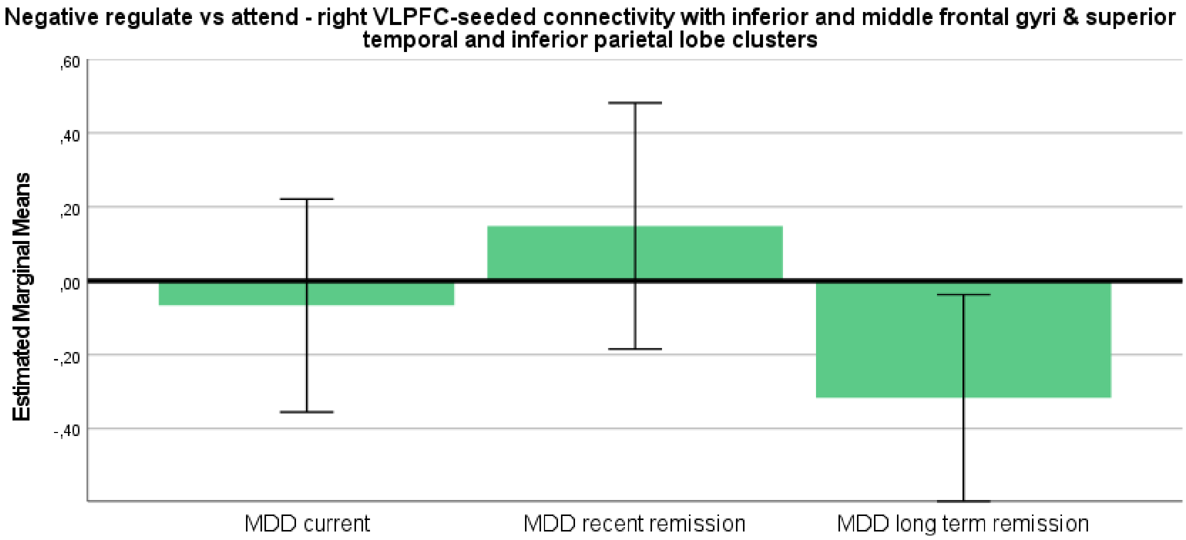

Supplementary Figure 5. Inferior and middle frontal gyri and superior temporal and inferior parietal lobe-clusters with which connectivity with the right VLPFC-seed was related to previous disease load during downregulation of negative emotions, including a plot of the estimated marginal means (plus 95% confidence intervals) of an ANCOVA on the contrast images within this cluster (including age, sex, education, scanner site and motion as covariates), split out per MDD subgroup.

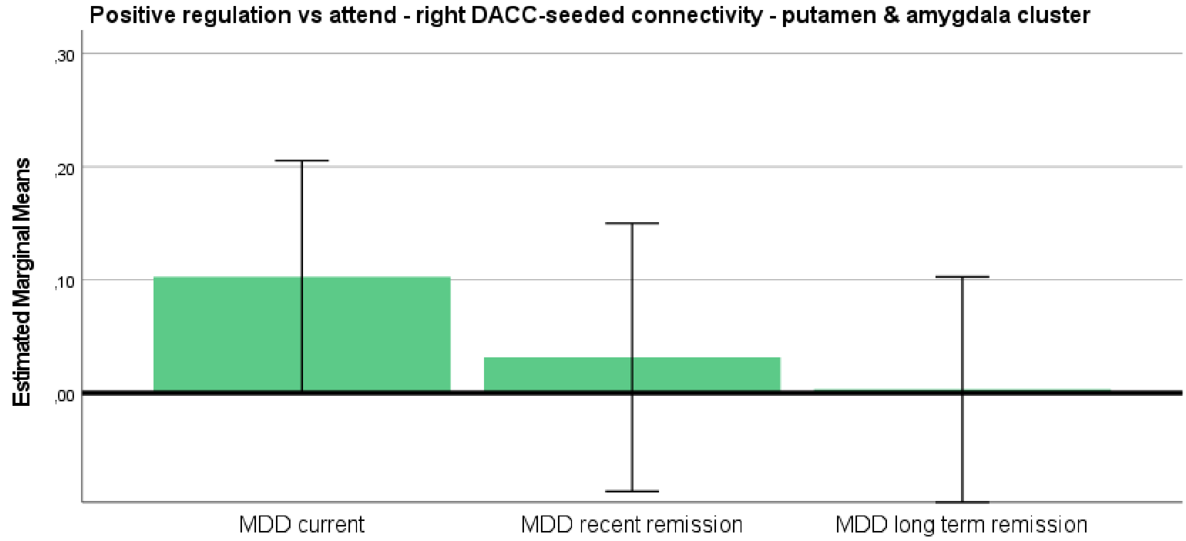

Supplementary Figure 6. Putamen and amygdala-clusters with which connectivity with the right DACC-seed was related to previous disease load during upregulation of positive emotions, including a plot of the estimated marginal means (plus 95% confidence intervals) of an ANCOVA on the contrast images within this cluster (including age, sex, education, scanner site and motion as covariates), split out per MDD subgroup.

Brain activity in MDD vs NDC

To further explore clinical state characteristics related to current vs remitted state, we additionally plotted the estimated marginal means of an ANCOVA on the contrast-values extracted from the significant clusters, split out for subgroups of patients (current depression, recent remission [1-6 month remission], long-term remission [>6 months]). Plots suggest that the SMA hypoactivity was strongest in the currently depressed patients.

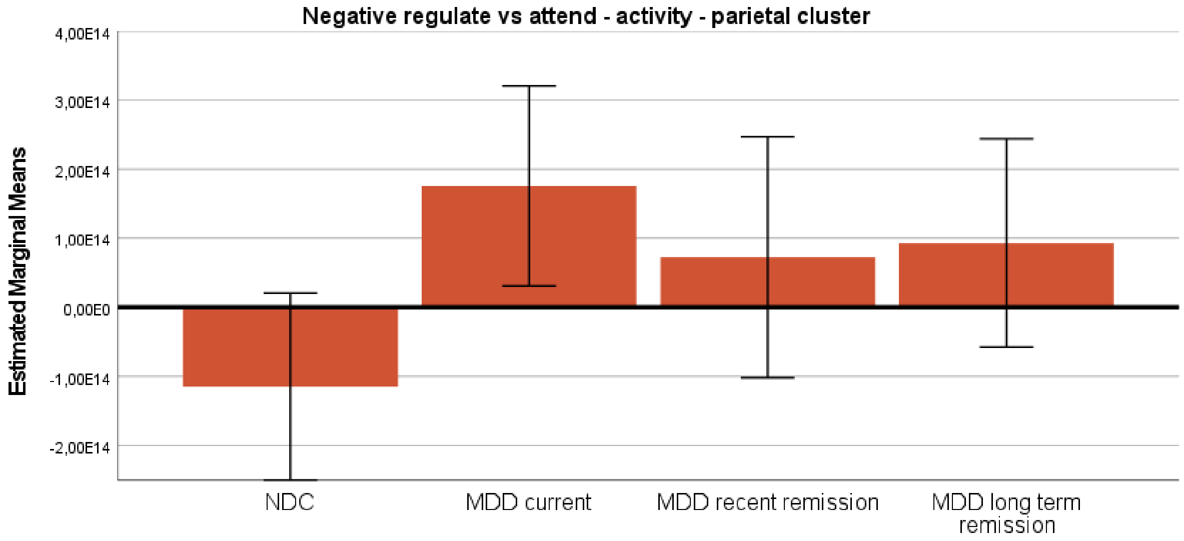

Supplementary Figure 7. Parietal clusters in which NDC showed significantly higher BOLD activation than MDD patients during downregulation of negative emotions, including a plot of the estimated marginal means (plus 95% confidence intervals) of an ANCOVA on the contrast images within this cluster (including age, sex, education, scanner site and motion as covariates), split out per MDD subgroup.

Brain connectivity in MDD vs NDC

Similarly, we plotted the estimated marginal means of an ANCOVA on the contrast-values extracted from the significant clusters, split out for subgroups of patients (current depression, recent remission [1-6 month remission], long-term remission [>6 months]). Plotting of these values suggested that the left dACC-, right dACC-, and right DLPFC-seeded connectivity abnormalities were observed independent of clinical state, while the right SMA-seeded connectivity abnormalities were less pronounced in the longer remitted (>1 year) depressed group.

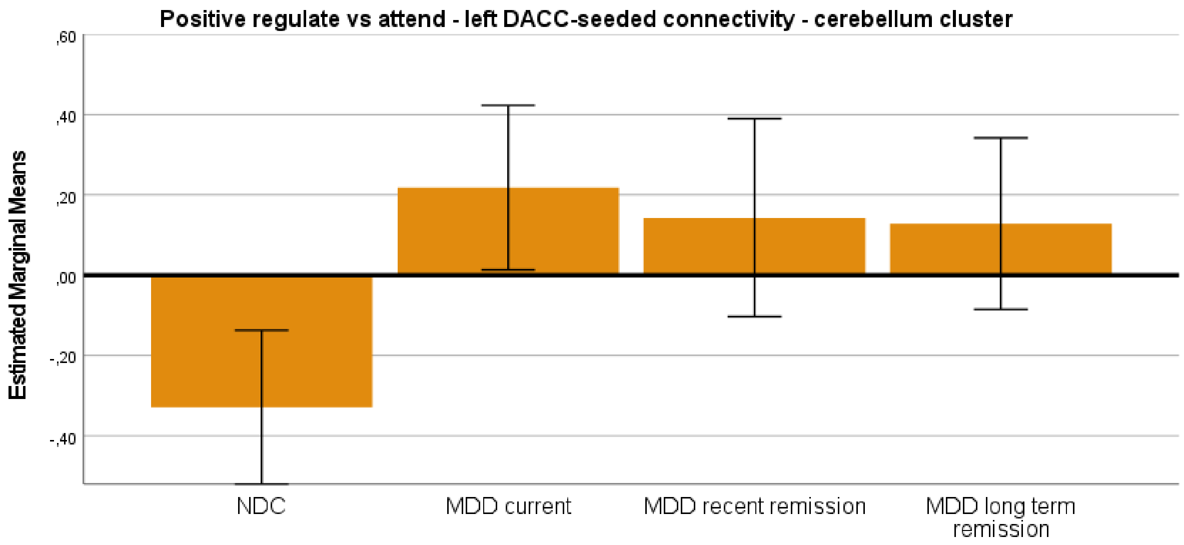

Supplementary Figure 8. Cerebellar cluster with which NDC showed significantly higher BOLD connectivity with the left DACC-seed than MDD patients during upregulation of positive emotions, including a plot of the estimated marginal means (plus 95% confidence intervals) of an ANCOVA on the contrast images within this cluster (including age, sex, education, scanner site and motion as covariates), split out per MDD subgroup.

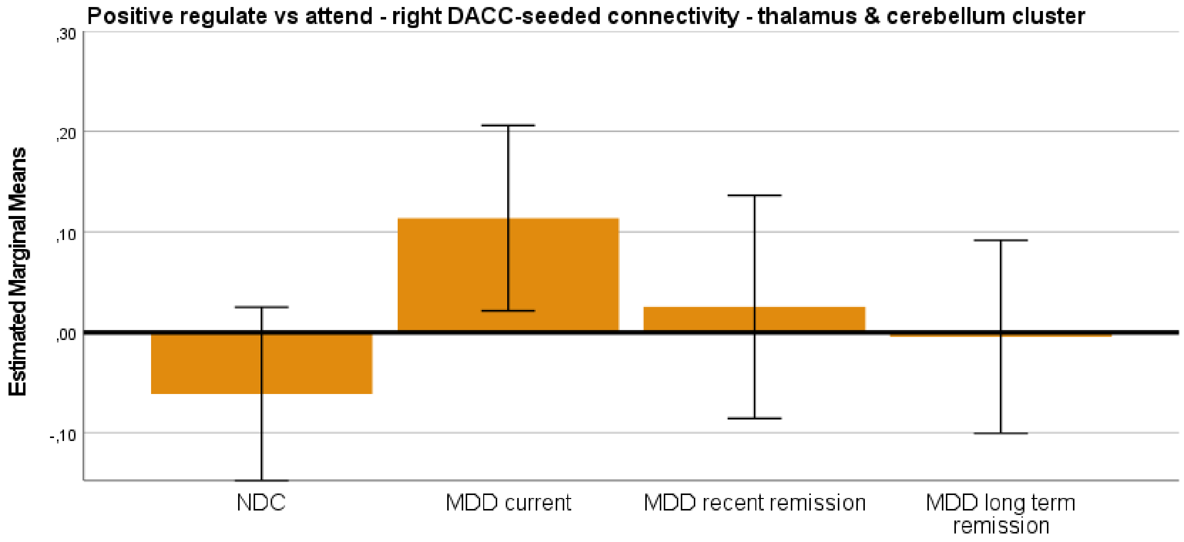

Supplementary Figure 9. Thalamus & cereballar cluster with which NDC showed significantly higher BOLD connectivity with the right DACC-seed than MDD patients during upregulation of positive emotions, including a plot of the estimated marginal means (plus 95% confidence intervals) of an ANCOVA on the contrast images within this cluster (including age, sex, education, scanner site and motion as covariates), split out per MDD subgroup.

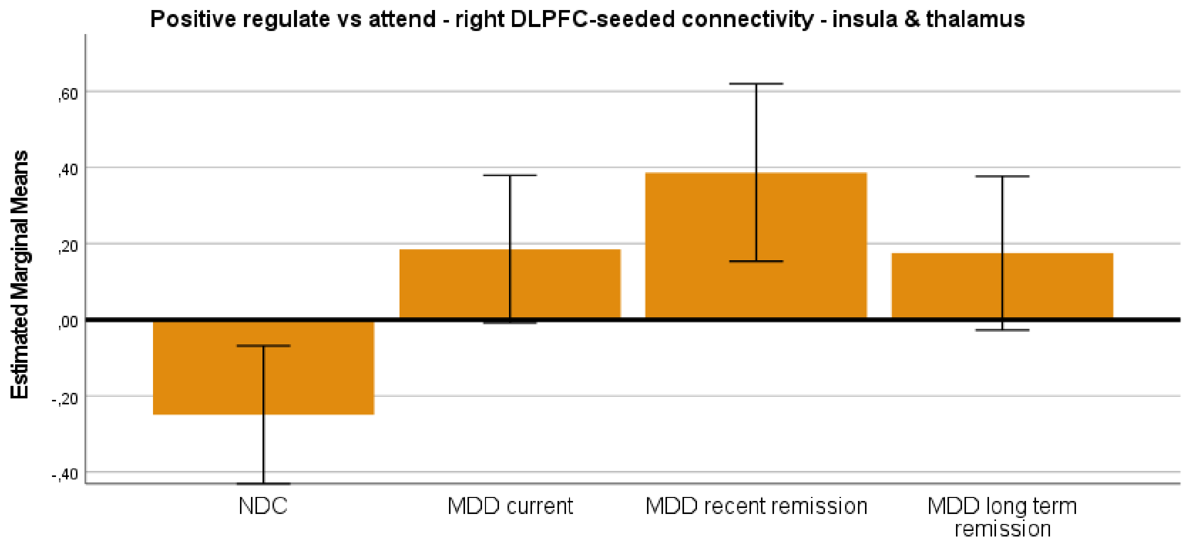

Supplementary Figure 10. Thalamus & insula-cluster with which NDC showed significantly higher BOLD connectivity with the right DLPFC-seed than MDD patients during upregulation of positive emotions, including a plot of the estimated marginal means (plus 95% confidence intervals) of an ANCOVA on the contrast images within this cluster (including age, sex, education, scanner site and motion as covariates), split out per MDD subgroup.

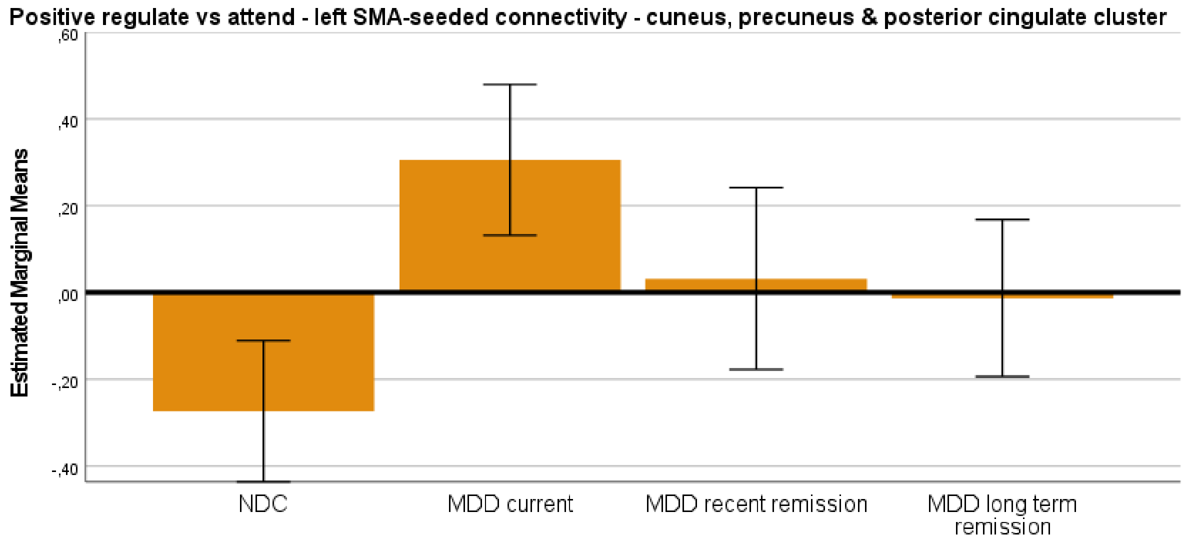

Supplementary Figure 11. Cuneus, precuneus and posterior cingulate cortex-cluster with which NDC showed significantly higher BOLD connectivity with the left SMA-seed than MDD patients during upregulation of positive emotions, including a plot of the estimated marginal means (plus 95% confidence intervals) of an ANCOVA on the contrast images within this cluster (including age, sex, education, scanner site and motion as covariates), split out per MDD subgroup.

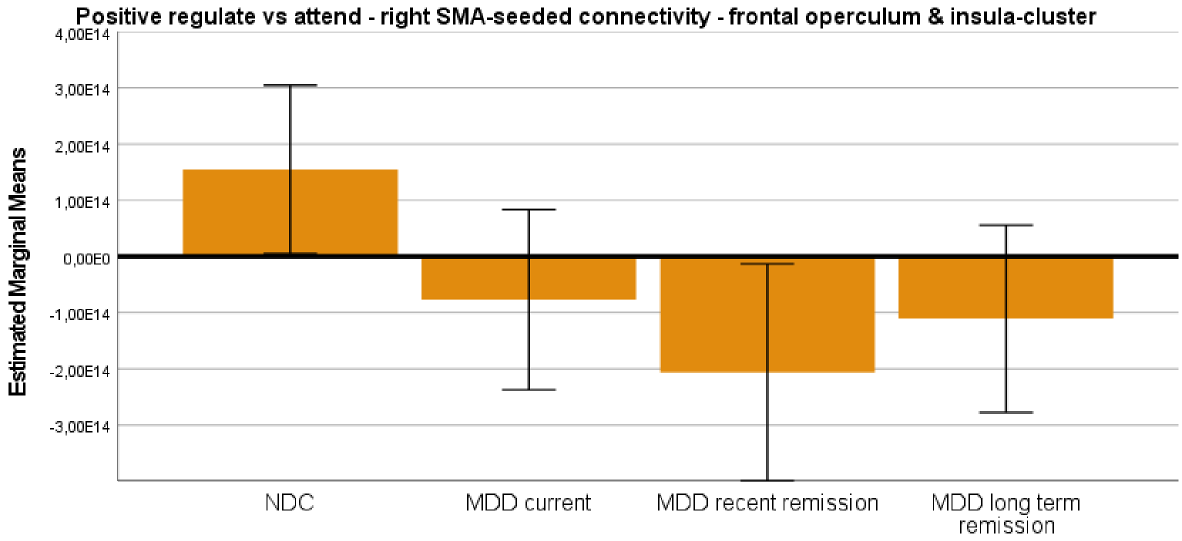

Supplementary Figure 12. Frontal operculum & insula-cluster with which NDC showed significantly higher BOLD connectivity with the right SMA-seed than MDD patients during upregulation of positive emotions, including a plot of the estimated marginal means (plus 95% confidence intervals) of an ANCOVA on the contrast images within this cluster (including age, sex, education, scanner site and motion as covariates), split out per MDD subgroup.

Supplement 11 Relation of duration and severity with functional connectivity results

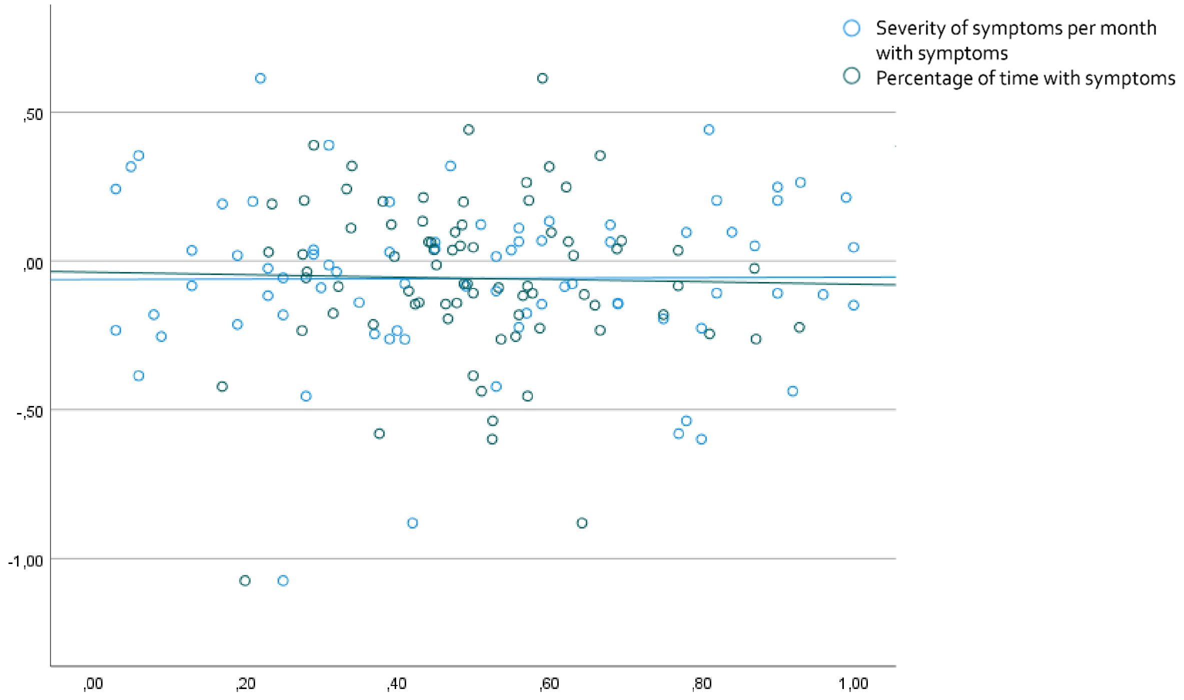

Supplementary Figure 13. Scatter plot of the relation between left amygdala seeded functional connectivity during downregulation of negative images (vs attend) and 1) severity of symptoms per month with symptoms (blue) and 2) percentage of time with symptoms of the total of months measured (green) in MDD patients, with fit lines.

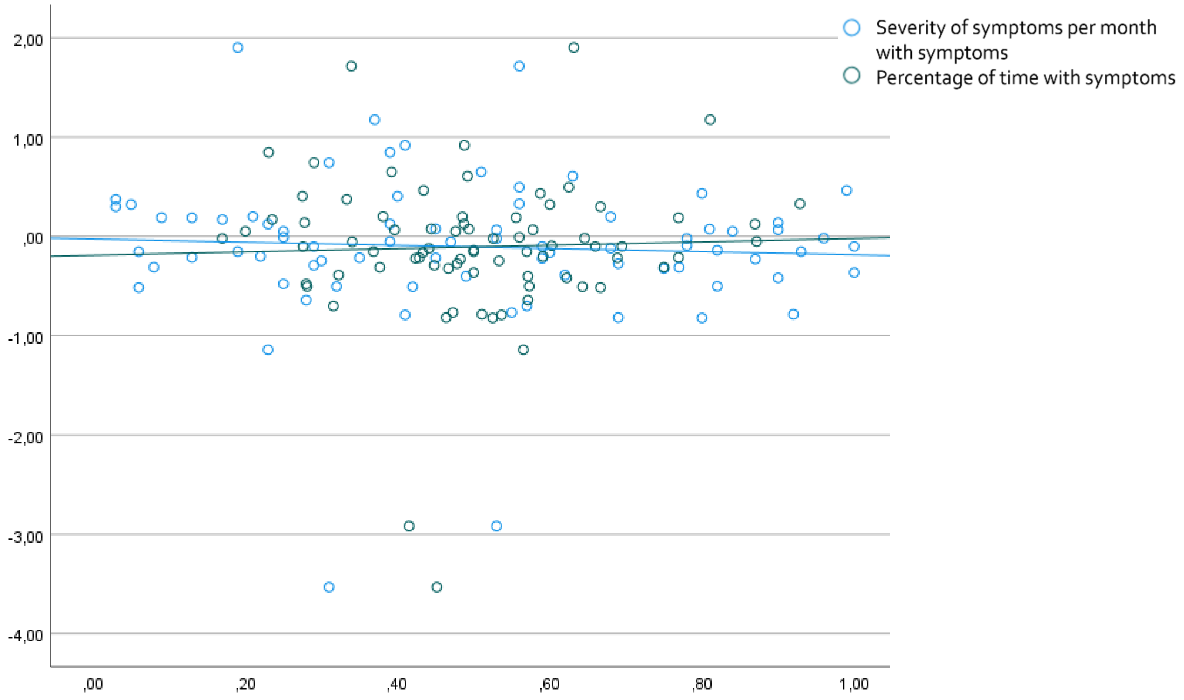

Supplementary Figure 14. Scatter plot of the relation between right VLPFC seeded functional connectivity during downregulation of negative images (vs attend) and 1) severity of symptoms per month with symptoms (blue) and 2) percentage of time with symptoms of the total of months measured (green) in MDD patients, with fit lines.

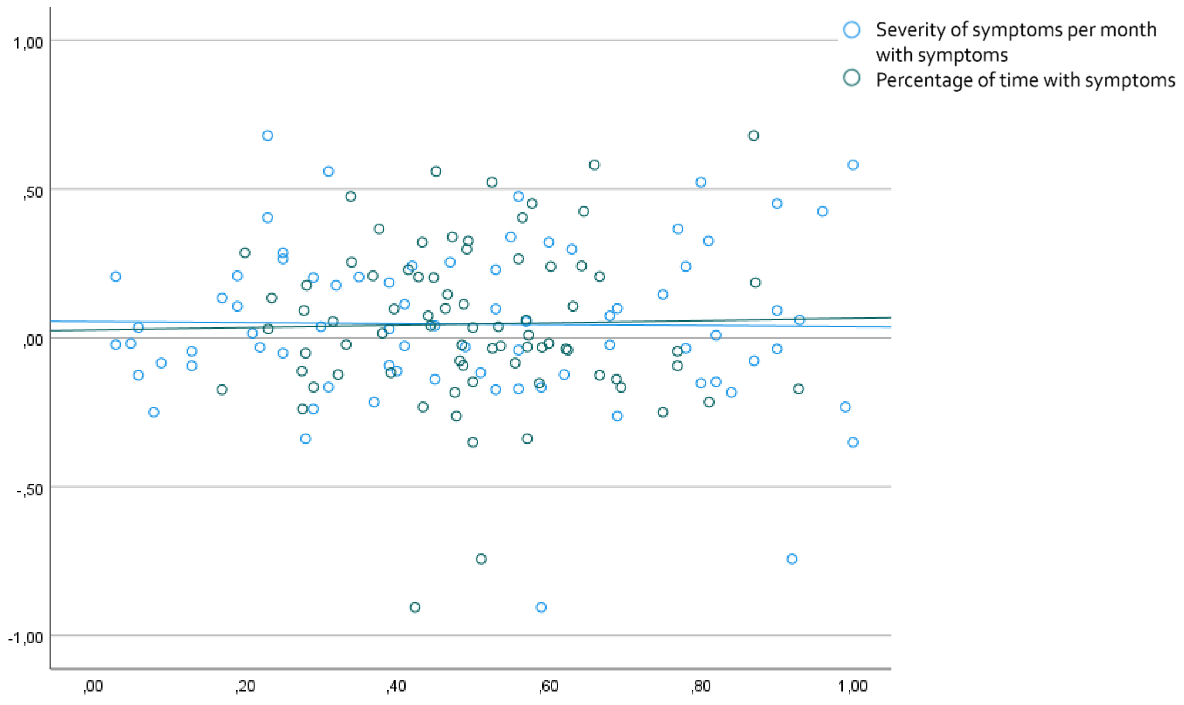

Supplementary Figure 15. Scatter plot of the relation between left DACC-seeded functional connectivity during upregulation of positive images (vs attend) and 1) severity of symptoms per month with symptoms (blue) and 2) percentage of time with symptoms of the total of months measured (green) in MDD patients, with fit lines.
